# The involvement of the *Candida glabrata* trehalase enzymes in stress resistance and gut colonization

**DOI:** 10.1080/21505594.2020.1868825

**Published:** 2020-12-28

**Authors:** Mieke Van Ende, Bea Timmermans, Giel Vanreppelen, Sofía Siscar-Lewin, Daniel Fischer, Stefanie Wijnants, Celia Lobo Romero, Saleh Yazdani, Ona Rogiers, Liesbeth Demuyser, Griet Van Zeebroeck, Yuke Cen, Karl Kuchler, Sascha Brunke, Patrick Van Dijck

**Affiliations:** a Laboratory of Molecular Cell Biology, Department of Biology, Institute of Botany and Microbiology, Leuven, KU Leuven, Belgium; b VIB-KU Leuven Center for Microbiology, Leuven, Belgium; c Department of Microbial Pathogenicity Mechanisms, Hans Knöll Institute, Jena, Germany; d Department of Internal Medicine and Pediatrics, Ghent University, Ghent, Belgium; e VIB-UGent Center for Inflammation Research, Ghent, VIB, Belgium; f Medical University of Vienna, Center for Medical Biochemistry, Max Perutz Labs Vienna, Campus Vienna Biocenter, Vienna, Austria

**Keywords:** *Candida glabrata*, trehalase, trehalose, colonization, stress, virulence

## Abstract

*Candida glabrata* is an opportunistic human fungal pathogen and is frequently present in the human microbiome. It has a high relative resistance to environmental stresses and several antifungal drugs. An important component involved in microbial stress tolerance is trehalose. In this work, we characterized the three *C. glabrata* trehalase enzymes Ath1, Nth1 and Nth2. Single, double and triple deletion strains were constructed and characterized both *in vitro* and *in vivo* to determine the role of these enzymes in virulence. Ath1 was found to be located in the periplasm and was essential for growth on trehalose as sole carbon source, while Nth1 on the other hand was important for oxidative stress resistance, an observation which was consistent by the lower survival rate of the *NTH1* deletion strain in human macrophages. No significant phenotype was observed for Nth2. The triple deletion strain was unable to establish a stable colonization of the gastrointestinal (GI) tract in mice indicating the importance of having trehalase activity for colonization in the gut.

## Introduction

Annually, invasive fungal infections cause 1.5 million deaths [1,2]. *Candida* species are among the most frequently isolated human fungi, with a mortality rate of up to 50% [1,3,4]. Of all *Candida* spp., *Candida albicans* is isolated most frequently [5]. However, the introduction of fluconazole as the first-line antifungal used in the clinic caused a decrease in the number of *C. albicans* infections and introduced an increase in infections caused by inherently more fluconazole tolerant species, such as *Candida glabrata* [5–8]. *C. glabrata* bloodstream infections are associated with high mortality rates and therefore, this fungus is becoming a major threat in hospitals [9,10]. The most important virulence factors of *C. glabrata* are its capacity to grow very rapidly at 37°C, adherence to various substrates and subsequent biofilm formation, its ability to intrinsically tolerate certain antifungal drugs, and its rapid adaptation to stresses [11–13]. In the human body, environmental stresses are ubiquitous, such as nutrient limitation and stress imposed by the host immune response. This activates the *C. glabrata* general stress response, which includes the induction of genes encoding enzymes involved in trehalose metabolism [14].

Trehalose is a non-reducing glucose disaccharide with a double role in fungi. It serves as a storage carbon source but is also crucial as a stress protectant molecule by stabilizing proteins and membranes, thereby causing resistance to antifungal drugs, oxidative stress, and heat stress [15–21]. The trehalose biosynthesis pathway is defined by two main enzymes: trehalose-6-phosphate synthase (Tps1) and trehalose-6-phosphate phosphatase (Tps2). Tps1 converts uridine diphosphate (UDP)-glucose and glucose-6-phosphate into trehalose-6-phosphate (T6P) and UDP, after which Tps2 hydrolyzes T6P into trehalose and free phosphate (Fig S1). Trehalose as well as the enzymes linked to its biosynthesis have been described to be involved in virulence and pathogenesis, with functions in infection, biofilm formation, etc [21–28]. Despite their absence in the human body and their long-known role in virulence, still no Tps1 or Tps2 inhibitor has been discovered and brought to clinical trials or to the clinic. As trehalose accumulates under stress conditions and thereby “freezes” proteins and membranes, a fast hydrolysis of trehalose upon relief of stress is essential to resume growth in *Saccharomyces cerevisiae* [29,30]. This hydrolysis is mediated by trehalase enzymes. Fungal species generally have two trehalase enzymes: a neutral and an acid trehalase enzyme. In the yeast model organism *S. cerevisiae*, the neutral trehalase enzyme *Sc*Nth1 is regulated by cAMP-dependent phosphorylation and ensures endogenous trehalose metabolization [31–33]. It requires divalent cations (Ca^2+^ and Mn^2+^) and has a pH optimum of 7 [34,35]. This neutral trehalase shows higher activity during exponential growth on fermentable carbon sources and is located in the cytoplasm of the cells [36]. On the other hand, fungal acid trehalase enzymes contain either a transmembrane domain or a signal peptide and are therefore located on the outside of the fungal cell; in the cell membrane, periplasm or cell wall [37–39]. The extracellular localization ensures hydrolysis of external trehalose [39–41]. In *S. cerevisiae*, the acid trehalase enzyme is also present in the vacuole [37,38]. The acid trehalase enzyme has an optimal pH of 4.5 and is not regulated by cAMP, phosphorylation or divalent cations [36].

Based on sequence homology with the *S. cerevisiae* trehalase enzymes, the *C. glabrata* genome encodes three trehalase enzymes: Ath1, Nth1, and Nth2. *C. glabrata* very rapidly hydrolyzes extracellular trehalose, a feature which is used in the clinic to diagnose *C. glabrata* infections in a quick and cost-effective way [42–48]. Recently, Ath1 (CAGL0K05137g) was found to be responsible for this extracellular trehalose fermentation [41]. The Ath1 orthologues in *C. albicans* (*CaATC1)* and *C. parapsilosis* (*CpATC1)* were found to be involved in virulence and stress resistance. The *CaATC1* and *CpATC1* deletion strains showed both an increased thermotolerance and an increased resistance toward oxidative stress [49–51]. Whereas these results would suggest an increase in virulence, this was not observed using a systemic mouse model, where these deletion strains showed a reduced virulence [49,51]. The *C. glabrata* neutral trehalases, Nth1 (CAGL0M10439g) and Nth2 (CAGL0C04323g) are considered to be cytosolic trehalases responsible for the hydrolysis of intracellular trehalose. In *C. albicans*, deletion of the neutral trehalase *CaNTC1* does not affect virulence in a mouse model of systemic infection [52].

Based on the importance of the trehalose metabolism for virulence in other pathogenic fungi and the observation that *C. glabrata* consumes extracellular trehalose very rapidly, we aimed to characterize the *C. glabrata* trehalase enzymes [49–51]. The trehalase deletion strains were first tested for *in vitro* growth on different relevant fermentable carbon sources. We confirm the data of Zilli, Lopes [41], where it was shown that Ath1 is required for utilization of exogenous trehalose. Moreover, we show that Ath1 is present in the periplasm. Hence, we hypothesized that the Ath1 enzyme could play a role in colonization of the human gut. We tested this in the gastrointestinal tract of mice observing the *C. glabrata* colonization over time. Unexpectedly, the *ath1*∆ mutant showed a wild type phenotype, whereas the triple deletion strain was unable to form a stable colonization of the GI tract. We also showed that *C. glabrata* Nth1 is involved in the oxidative stress response. As *C. glabrata* has the capability to survive and replicate in immune cells, the trehalase mutant strains were tested for survival in human macrophages. After four days, the single deletion strains and the triple deletion strain showed a reduced survival in the macrophages compared to the wild type. Finally, we tested the trehalase deletion strains in a mouse model of systemic infection. Only minor differences were observed between the different strains with a small increase in mortality rate for the *nth1∆* strain. Together, these results indicate that the trehalase enzymes could be a good target for the development of new antifungal drugs, especially for their colonization in the gut. As we expect that targeting of one trehalase is insufficient, we suggest to target all three trehalase enzymes with a competitive inhibitor.

## Materials and methods

### Construction of phylogenetic tree

To create the trehalase phylogenetic tree, protein sequences were retrieved from the *Candida* Genome Database (CGD), *Saccharomyces* Genome Database (SGD), *Aspergillus* Genome Database (AspGD), UniProt, and PomBase [53–57]. In CLC Main Workbench, protein sequences were aligned and the phylogenetic tree was constructed using the unweighted pair method with arithmetic mean (UPGMA) and the Kimura Protein as a protein distance measure [58].

### Yeast strains, plasmids, primers and media

The yeast strains, plasmids and primers used in this study are listed in Supplementary Table 1. Yeast cells were grown in either YP (1% yeast extract, 2% bacteriological peptone), synthetic complete (SC) medium (1.7 g/L Difco yeast nitrogen base without amino acids and without ammonium sulfate, 0.79 g/L complete supplement mixture [CSM; MP Biomedicals], 5 g/L ammonium sulfate, pH 5.5 (liquid) or 6.5 (solid)), YNB (1.7 g/L Difco yeast nitrogen base without amino acids and without ammonium sulfate, 20 mg/L tryptophan, 20 mg/L histidine, 30 mg/L leucine, 5 g/L ammonium sulfate, pH 5.5 (liquid) or 6.5 (solid)) or RPMI 1640 medium. These media were supplemented with glucose or trehalose as indicated in the experiment. For solid media, 15 g/L Difco agar granulated was added.

### Construction of plasmids

For expression of the flippase enzyme, the nourseothricin cassette of vector pLS9 was replaced by the hygromycin marker resulting in pLS10. The hygromycin marker was amplified using primers C6240 and C6241 from plasmid pgRNA-uni-hph (p58) and subsequently ligated into the *Not*I digested pLS9. Colonies were checked using primers 7883 and B3220 and verified by sequencing. For localization experiments, the trehalase enzymes were fused to mCherry. Therefore, open reading frames (ORFs) were amplified using primers D84 and D1728 (*ATH1*), D88 and D89 (*NTH1*), or D92 and D93 (*NTH2*) from wild type genomic DNA. The terminators were amplified using primers D86 and D87 (*ATH1*), D90 and D91 (*NTH1*), or D94 and D95 (*NTH2)* from wild type genomic DNA. The corresponding ORF and terminator fragments were inserted in *BamH*I and *Xho*I digested pYC56 vector using NEBuilder (New England Biolabs), resulting in the expression vectors pBM13 (*ATH1*), pBM14 (*NTH1*), and pBM15 (*NTH2*). Correct insertion of the ORF fragments was checked using primers C2950 and A9050, insertion of terminator fragments was checked using primers B1222 and A2047.

### *Construction of* C. glabrata *strains*

The trehalase deletion strains were constructed in the ATCC2001 *his3∆ trp1∆ leu2∆* background (Fig S2) [59]. The wild type strain was transformed by electroporation with the deletion cassette (a nourseothricin cassette flanked by FRT sites and a 100 bp region flanking the targeted gene). The deletion cassettes were PCR amplified from the pYC44 plasmid using primers C6315 and C6316 (*ATH1*), C6317 and C6318 (*NTH1*) or C6319 and C6320 (*NTH2*). Cells were plated on YPD agar medium supplemented with 200 µg/mL nourseothricin. Transformants were checked for insertion of the deletion cassette by PCR using control primers C3177 and C3178 (*ATH1*), C3183 and C3184 (*NTH1*) or C3161 and B3162 (*NTH2*). Correct strains were subsequently transformed with plasmid pLS10 to induce expression of the flippase enzyme (300 µg/ml hygromycin selection). Removal of the nourseothricin cassettes of the transformants was checked by PCR using primers C3177 and C8497 (*ATH1*), C3183 and C3835 (*NTH1*) or C3161 and B2011 (*NTH2*). The pLS10 plasmid was lost by growth on nonselective YPD medium and checked by replating on YPD supplemented with 300 µg/mL hygromycin. The trehalase double and triple mutants were constructed similarly, by transformation of the nourseothricin deletion cassettes mentioned above in the single or double deletion strains, respectively, followed by removal of the selective marker by expression of pLS10. Endogenously tagged trehalase strains were constructed by electroporation of the wild type strain with *Eci*I digested plasmid pBM13 (*ATH1*-mCherry), pBM14 (*NTH1*-mCherry), or pBM15 (*NTH2*-mCherry). The resulting transformants were checked using primers C9381 (*ATH1*), C9385 (*NTH1*) or C9388 (*NTH2*), and A9050 (mCherry).

### Growth phenotype

Growth of the strains was followed in time both in liquid and on solid YNB medium over time. Overnight cultures of the different strains were washed three times with sterile milli-Q water and subsequently diluted to an OD_600_ of 0.1. For growth assays in liquid medium, cells were grown in 50 mL YNB containing either 10 mM of glucose or 10 mM of trehalose as the carbon source. The cells were grown at 37°C with continuous shaking at 200 rpm for 72 hours during which the OD_600_ was monitored. For growth assays on solid medium, a tenfold dilution series of the cultures was spotted on YNB plates containing 5 mM of glucose or 5 mM of trehalose. To investigate the stress response, the washed overnight cultures were spotted on YNB plates supplemented with 100 mM glucose, containing either 6 mM H_2_O_2_, 1.5 M NaCl or 0.4 mg/mL CFW. The plates were incubated at 37°C for 72 hours during which growth was assessed.

### Protoplast preparation

Overnight cultures of the different strains were made in YPD medium. Subsequently, the cultures were grown until mid-exponential phase in YPD (100 mM glucose) or YPT (100 mM trehalose). The cells were collected and washed twice, after which they were incubated for 15 to 45 minutes in the protoplasting solution (600 mM KCl, 800 mM Sorbitol, 41.7 mM K_2_HPO_4_, 8.3 mM KH_2_PO_4_, 4.8 units/mL Zymolase, 9.65 mM β-mercapto-ethanol) until the OD_600_ dropped. The cells were collected and washed twice with cold protoplasting buffer (166.8 mM K_2_HPO_4_, 33.2 mM KH_2_PO_4_, 800 mM Sorbitol), after which trehalase activity was determined as described below.

### Determination of trehalase activity

Trehalase activity was determined as described in Pernambuco, 1996 [60]. In short, crude extracts were incubated with 50 µL of acid trehalose buffer (50 mM trehalose, 200 mM sodium citrate, 2 mM EDTA, pH 4.5). After 30 minutes of incubation at 30°C, the reaction was terminated by boiling for 5 minutes at 90°C. The glucose liberated was determined by the glucose oxidase–peroxidase method. Protein levels were determined by the Lowry method. Trehalase specific activity is expressed as nmol of glucose released per min and mg of protein.

### Determination of extracellular compounds

Extracellular glucose and trehalose concentrations during growth were analyzed by the Shimadzu HPLC system using an Agilent 87 H column at 0.7 mL/min and a RID-20A detector (Shimadzu).

### Fluorescence microscopy

We used a FluoView FV1000 confocal microscope (Olympus IX81) and its software for localization of Ath1. We visualized mCherry with a 559-nm laser and BA575-675 emission filter. A 60x UPlanSApo (numerical aperture [NA], 1.35) objective lens was used.

### Acute hydrogen peroxide survival

Overnight cultures of the strains in SC 100 mM glucose were inoculated in 50 mL SC 100 mM. These cultures were allowed to grow for 48 hours at 37°C until reaching stationary phase. The cells were washed and diluted to OD_600_ 0,5 in YNB 100 mM containing different concentrations of H_2_O_2_ (0, 6, 10, 20, 50, and 100 mM). The cells were kept under stress conditions for 1 h at 37°C after which they were washed with water. The cells were diluted fivefold and spotted onto YPD plates which were placed at 37°C. Scans of survival were taken after 24 hours.

### Ethical statement

Blood was obtained from healthy human volunteers with written informed consent according to the declaration of Helsinki. The blood donation protocol and use of blood for this study were approved by the Jena institutional ethics committee (Ethik-Kommission des Universitätsklinikums Jena, Permission No 2207–01/08).

## Expression analysis

### Short term analysis in murine cell line macrophages (RAW264.7)

5 X 10^6^ RAW264.7 cells (murine macrophage cell line) were seeded in 14 mL DMEM + 10% FBS (PAA laboratories) into 100 mm diameter cell culture petri dishes (TPP, Techno Plastic Products) and incubated for two days with a medium exchange after one day. A *C. glabrata* ATCC2001 overnight culture was pelleted, taken up in DMEM + 100 µg/mL Ampicillin + 100 µg/mL Kanamycin and cell counts were determined. 108 *C. glabrata* yeast cells were added per petri dish and infected petri dishes were stored immediately for 30 min on ice for synchronization of phagocytosis. After 30 min, non-adhered yeast were washed away twice with PBS after which 14 mL DMEM + 100 µg/mL Ampicillin + 100 µg/mL Kanamycin was added and co-incubation at 37°C and 5% CO2 was started. Samples were taken after 0 (directly from ice), 10, 30, 60, 180, and 360 min: non-phagocytosed yeast were washed away twice with PBS and macrophages were lysed with AE buffer + 1% SDS. Centrifugation (2 min 12.000 g) was used for separation of the fungal cell pellet from macrophagal DNA and RNA. The fungal cell pellet was frozen in liquid nitrogen.

### Long-term analysis in human monocyte-derived macrophages (hMDMs)

Method details are described in Fischer et al. (in preparation). Briefly, hMDM monolayers in RPMI + 10% HS were infected in cell culture flasks (Greiner) with *C. glabrata* ATCC2001 at an MOI of 20 and non-phagocytosed yeasts were washed away with PBS twice after 3 hours of co-incubation. Caspofungin was added after 6 hours of co-incubation and further held constant on a level of 5 µg/mL to hinder extramacrophagal yeast growth. Medium was in part exchanged daily. Samples were taken after 0,25, 1, 2, and 4 days widely similar as above (but with AE + 10% SDS).

### RNA isolation, labeling and microarray analysis

Fungal RNA was isolated using a modified freeze-thaw protocol [61]. Optionally, β-mercaptoethanol was used at a final concentration of ≈ 5% in AE buffer for yeast resuspension. The QuickAmp Labeling Kit (Agilent) was used to generate Cy5-labeled cRNA (Cy5 CTP; GE Healthcare). Cy5-labeled samples were co-hybridized with a Cy3-labeled reference (RNA isolated from *C. glabrata* ATCC2001 grown to mid-log phase) on 8-by-15 K format arrays (Agilent) and scanned either in Agilent´s High Resolution C Scanner with Scan Control (Agilent) or in a GenePix 4200AL with GenePix Pro 6.1 (Auto PMT, pixel size 5 µm). Microarray data were analyzed using GeneSpring 14.8 (Agilent).

### Fungal killing by human macrophages

#### Differentiation of human monocytes into human monocyte-derived macrophages

Preparation of human monocyte-derived macrophages (hMDMs) was done as described previously [62]. Briefly, monocytes were selected from buffy coats by magnetic automated cell sorting of CD14 positive monocytes, seeded in 175 cm^2^ cell culture flasks (Greiner) and differentiated over a time period of seven days. At day 7, hMDMs were detached, and 1.5 × 10^5^ cells/well were seeded for infection in a 24-well plate in RPMI + 10% FBS (Gibco). The day after, the medium was exchanged to RPMI + 10% human serum (HS; from AB male donors; sterile-filtered, Bio&Sell) with an intermediate PBS washing step.

#### Macrophage infection with C. glabrata

For the preparation of yeast inocula, overnight cultures of *C. glabrata* strains were pelleted, washed twice with PBS and adjusted to 1.5 × 10^6^ cells/mL. The macrophages were infected with 1.5 × 10^5^ *C. glabrata* cells (multiplicity of infection (MOI) 1:1). Three hours after infection, the non-phagocyted cells were washed away and plated to check for phagocytosis efficiency and 1 mL of fresh RPMI + 10% HS was added per well. At this same time point, also the lysate was plated for the 3 h timepoint. At one and four days, the supernatant was removed without washing, and the hMDMs lysed and plated. From wells intended for four days co-incubation, 0.5 mL of supernatant was removed after one day and 1 mL fresh medium was added (total volume 1.5 mL). On each following day, 0.5 mL was replaced with fresh medium. All wells were constantly checked for appearance of yeast microcolonies by naked eye. Before macrophage lysis and yeast plating, each well was systematically checked for extracellular yeasts with an inverse microscope, and only wells that met the cutoff criteria were used for plating. CFU counts on YPD agar plates were determined manually.

### In vivo *mouse model of systemic infection*

The virulence of the different strains was assessed in an *in vivo* mouse model of systemic infection in female BALB/c mice (8 weeks old). The mice were housed in groups of four in filter-top cages in a dedicated animal room where temperature, light, and humidity were regulated. The animals received a standard *ad libitum* diet and water. At day −3, all mice were immunosuppressed with 75 mg/kg dexamethasone (Fagron) through intraperitoneal injection. This concentration was chosen after a pilot study as the effect of the trehalase deletion on the infection can be assessed (more or less virulent). After this, the animals received the same amount of immunosuppression on day 0 and from then on, every seven days. On day 0, the mice were injected intravenously via the lateral tail vein with 5 × 10^7^ *C. glabrata* cells in 200 µL PBS. After this, the infected mice were monitored daily and when they reached humane endpoints, they were euthanized by cervical dislocation under anesthesia. The survival assay was terminated at day 18 after infection.

### In vivo *mouse model of gastrointestinal colonization*

The gut colonization capacity of the different strains was determined in an *in vivo* mouse model of gastrointestinal colonization in female c57BL6/J mice (8 weeks old). The mice were housed in groups of four in filter-top cages in a dedicated animal room where temperature, light and humidity were regulated. The animals received a standard *ad libitum* diet and water. At day 0, the mice received 10^8^ *C. glabrata* cells via oral gavage. From this day on, stool samples from each mouse were collected during 21 days at the timepoints indicated and plated on CHROMagar^TM^ for CFU counting. The GI colonization is expressed as log(CFU/gram of stool). The gastrointestinal colonization model was terminated at day 21 after gavage. The animals were sacrificed by cervical dislocation under anesthesia and duodenum, ileum, cecum, proximal colon, distal colon, tongue and kidneys were plated for CFU counting on CHROMagar^TM^.

## Results

### C. glabrata *trehalase enzymes are phylogenetically closely related to* S. cerevisiae *trehalase enzymes*

**Figure 1.**
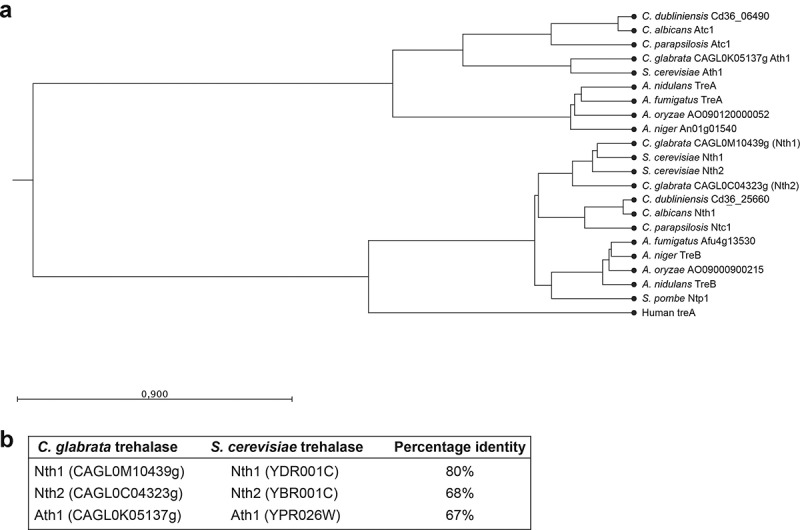
**Phylogenetic tree of fungal trehalases**. Two main clusters can be distinguished: the neutral trehalases and the acid trehalases. The human trehalase enzyme was included and is present in the cluster of neutral trehalase enzymes. Protein sequences were aligned and the phylogenetic tree was constructed using the unweighted pair method with arithmetic mean (UPGMA) and the Kimura Protein as a protein distance measure (a). Overview of amino acid identity between the *C. glabrata* and *S. cerevisiae* trehalases (b)

Despite what its name suggests, *C. glabrata* is more related to species within the *Saccharomycetaceae* clade than to species within the *Candida* clade [63,64]. Therefore, we used the *S. cerevisiae* trehalase protein sequences in a BLASTP search to identify the *C. glabrata* trehalase enzymes. In this manuscript, genes/proteins of other species than *C. glabrata* are indicated with a prefix. For the neutral trehalases *Sc*Nth1 (YDR001C) and *Sc*Nth2 (YBR001C), the *C. glabrata* Nth1 (CAGL0M10439g) and Nth2 (CAGL0C04323g) were identified as closest orthologs respectively. These proteins are highly conserved to their *S. cerevisiae* orthologs, as reflected by the high percentage of amino acid identity (80% for Nth1 and 68% for Nth2) (Figure 1b). As is the case for *S. cerevisiae*, Nth1 and Nth2 are highly similar with one another (71% amino acid identity), indicating that these enzymes are most probably the result of the whole genome duplication event [65]. *NTH1* encodes for a polypeptide of 769 amino acids with a molecular mass of 87.4 kDa, while *NTH2* encodes for a polypeptide of 750 amino acids with a molecular mass of 86.5 kDa. Both Nth1 and Nth2 amino acid sequences contain neither a transmembrane domain nor a signal sequence [66,67]. Alignment of Nth1 with *Ca*Nth1 (CR_00560W_A) shows 58% amino acid identity, reflecting the more distant phylogenetic relationship between *C. glabrata* and *C. albicans.*

For the acid trehalase *Sc*Ath1 (YPR026W), Ath1 (CAGL0K05137g) was found as an ortholog in *C. glabrata*, sharing 67% amino acid identity. *ATH1* encodes for a polypeptide of 1212 amino acids and 136.5 kDa. In contrast, alignment of Ath1 with *Ca*Atc1 (C1_06940C_A) showed only 41% amino acid identity (Figure 1b). In other organisms, Ath1 was often found on the extracellular side of the cells [37,39,40,51,68,69]. Hence, the PROTTER and the SignalP4.1 algorithms were used to predict a possible transmembrane domain or a signal sequence [66,67]. No signal peptide was found, but Ath1 is predicted to contain one transmembrane (TM) domain at the N-terminus (between positions 83 and 103). The orthologous N-terminus and TM domain of *Sc*Ath1 confers the extracellular localization, suggesting identical extracellular localization for Ath1 in *C. glabrata* [37].

To represent the evolutionary relationship, we constructed a phylogenetic tree of the different fungal trehalase enzymes and also included the human trehalase enzyme (Figure 1a). Two main clusters can be distinguished: the neutral trehalases and the acid trehalases. The *C. glabrata* neutral trehalases Nth1 and Nth2 are closely related to *S. cerevisiae Sc*Nth1 and *Sc*Nth2 respectively. The acid trehalase Ath1 is most related to *Sc*Ath1. Again, this is not unexpected as these species share a close evolutionary relationship [63,64].

### Ath1 is required for growth on trehalose as a carbon source

**Figure 2.**
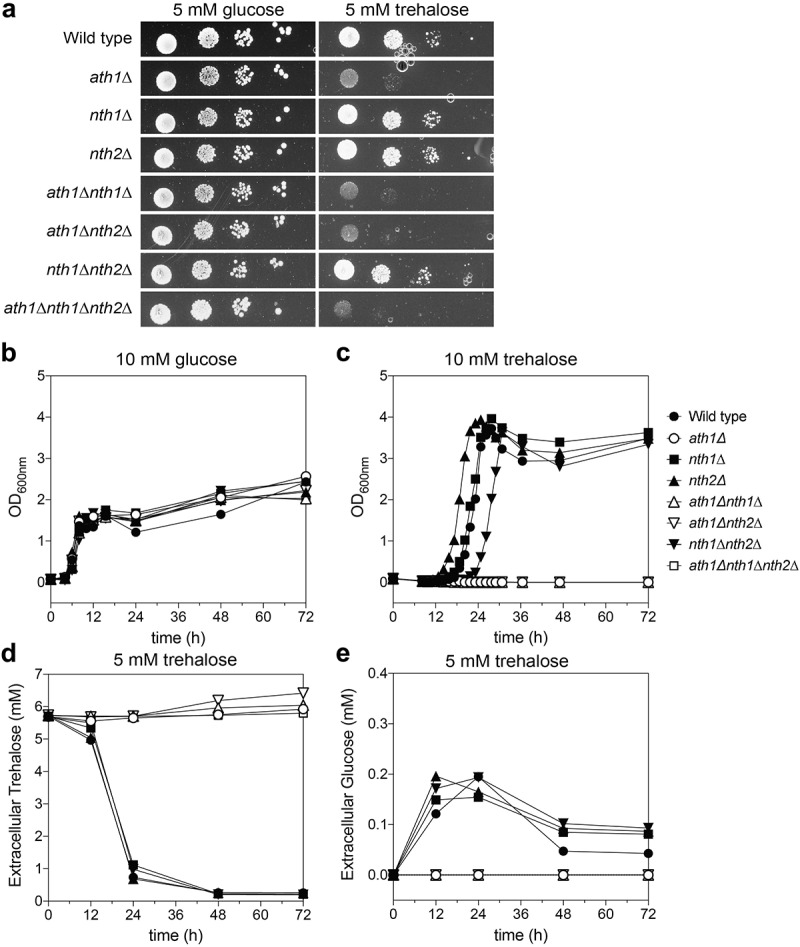
**Growth of the different trehalase deletion strains**. Cells were grown on YNB solid (a) and in liquid (b-e) medium containing glucose (a-b) or trehalose (A, C-E) as a fermentable carbon source. During growth in YNB trehalose, we also measured the trehalose and glucose concentrations in the medium over time (D-E, respectively). The experiments were performed at least twice and average results of three independent transformants are shown

**Figure 3.**
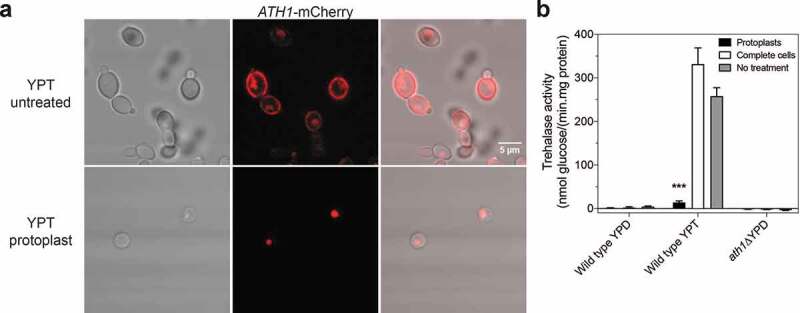
**Ath1 is present in the periplasm and is responsible for high trehalase activity when cells are grown in presence of trehalose**. Imaging of Ath1-mCherry cells taken with a Fluoview 1000 confocal microscope (a). Cells were grown in presence of trehalose to exponential phase after which protoplasts were made. The upper panel of A shows complete cells, the lower panel shows protoplasts of Ath1-mCherry. The trehalase activity was measured in complete cells, protoplasts and cells with no treatment after growth on YPD or YPT (b). Average trehalase activity of at least two experiments is shown with the standard error of the mean (SEM). Statistical Kruskal-Wallis test was used with Dunn’s correction for comparing treated and untreated conditions for each strain (*, P ≤ 0,05; **, P ≤ 0,01; *** P ≤ 0,001 and ****, P ≤ 0,0001)

In order to study the *C. glabrata* trehalase enzymes, we generated the single-, double- and triple trehalase deletion strains as described in Materials and Methods. As a first step of the *in vitro* characterization of the constructed mutants, we tested the growth of the strains in minimal medium supplemented with glucose or trehalose, two carbon sources that *C. glabrata* is able to ferment [70]. All strains grew to the same extent on glucose in both solid and liquid medium (Figure 2(a,b)). When trehalose was used as external carbon source, the growth of all strains lacking *ATH1* had a growth defect in both liquid and solid medium (Figure 2(a,c)). These results indicate that Ath1 is responsible for the hydrolysis of exogenous trehalose, which led to two hypotheses: either *C. glabrata* transports extracellular trehalose to the inside of the cell by a trehalose transporter and Ath1 hydrolyzes the disaccharide intracellularly, or Ath1 is present at the extracellular side of the cells and hydrolyzes trehalose in the surrounding medium after which glucose is taken up by the glucose transporters. In *S. cerevisiae*, extracellular trehalose is transported by the high-affinity sugar transporter *Sc*Agt1 [71]. Therefore, a BLASTP search was performed using *Sc*Agt1, however no orthologs could be identified in *C. glabrata*. Hence, we measured the concentration of trehalose in the medium over time during the growth on trehalose. We postulated that if Ath1 does indeed hydrolyze trehalose intracellularly after uptake by transporters, a strain deleted for *ATH1* would still show reducing extracellular trehalose levels over time. Yet, for the strains with a deletion of *ATH1*, the trehalose in the medium remained constant (Figure 2d). In contrast, the strains with wild type *ATH1* could still hydrolyze the extracellular trehalose, which is reflected by a drop of trehalose in the medium (Figure 2d). Additionally, coinciding with this decrease in trehalose, a small increase of glucose in the medium was observed, confirming extracellular hydrolysis of trehalose in two glucose molecules (Figure 2e). Taken together, these results suggest that Ath1 hydrolyses extracellular trehalose and thus indicate that Ath1 is present at the cell surface or is secreted. Therefore, we determined its localization by tagging Ath1 endogenously with a fluorescent protein, mCherry. When *C. glabrata* was grown in glucose-containing medium to the exponential phase, no fluorescence was detected. However, when trehalose was used as a carbon source, a signal on the border and in the vacuole of the cells was observed (Figure 3a and Fig S3). Next, we investigated whether Ath1 is anchored in the plasma membrane as Ath1 contains one predicted TM domain. Therefore, we generated protoplasts of cells grown in glucose or trehalose containing medium and measured the trehalase activity. The basal activity of the cells grown on glucose is very low and is not affected by protoplast formation. When grown on trehalose, the high trehalase activity observed in untreated cells, dropped dramatically in protoplasts (Figure 3b). In addition, the fluorescent signal observed on the outside of untreated cells, was no longer visible in protoplasts (Figure 3a). These results indicate that Ath1 is not anchored to the cell membrane but is rather present in the periplasmic space or in the cell wall.

### The triple deletion strain is unable to stably colonize the gut

**Figure 4.**
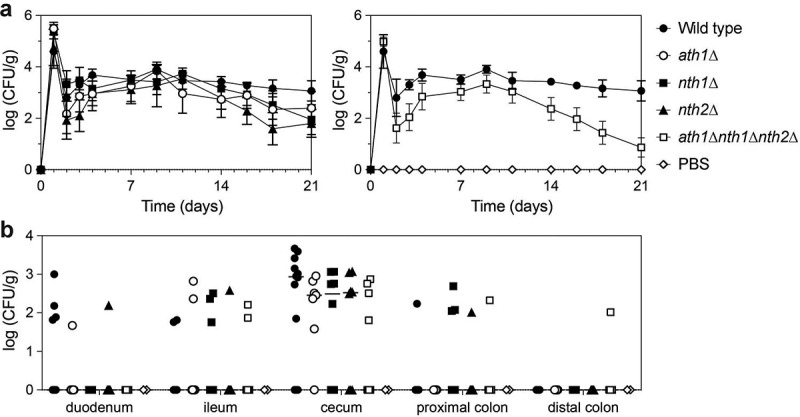
**Colonization in the GI tract is not maintained in the triple trehalase deletion strain**. At day 0, mice are orally infected with 10^8^ *C. glabrata* cells (gavage). Wild type (●), *ath1*∆ (○), *nth1*∆ (■), *nth2*∆ (▲) and *ath1∆nth1∆nth2∆* (Δ) or PBS as a control (□) were tested. At the indicated days a stool sample was collected from each mouse and plated for CFU counting, during 21 days (a). Upon termination of the experiment, mice were sacrificed and different tissues were collected to plate for CFU counting (b). All data are expressed in Log10 values

As humans cannot produce trehalose, its presence in the gut originates from ingested food or from micro-organisms as a result of production or release after microbial death. As Ath1 is required for growth on extracellular trehalose, we hypothesized that this enzyme could give *C. glabrata* cells a competitive advantage over other micro-organisms. To test this hypothesis, we verified whether the wild type and different deletion strains were able to colonize the gut over a longer period of time in a mouse model. To achieve *Candida* colonization in the gut, mice are frequently treated with antibiotics [72,73]. As we want to assess the competition with these other micro-organisms, a pilot experiment was performed in non-treated mice and mice which received ampicillin in their drinking water (1 mg/ml) infecting them via oral gavage with wild type *C. glabrata* (Fig S4). Despite the higher colonization in mice receiving ampicillin, we also obtained a stable colonization in mice where microbial perturbation was not induced (Fig S4). Because of the higher relevance of the latter model, we continued the experiments in untreated mice and tested the trehalase single deletion strains and the triple deletion strain (Figure 4). Similar to the pilot experiment, a stable colonization was obtained for the wild type strain (Figure 4a). The main habitat of *Candida* in the GI tract was in the cecum, which had a log (CFU/g) of around 3 (Figure 4b). The single deletion strains showed a similar colonization as the wild type strain (Figure 4a). The triple deletion strain was not able to establish a stable colonization of the GI-tract, as over 66% of the mice cleared the *C. glabrata* administered at day 21. This trend is clearly reflected in the right panel of Figure 4a. Logically, the average colonization of the cecum of mice which were orally infected with the triple deletion strain was significantly lower, as over 66% of the mice showed no colonies (Figure 4b).

### *Nth1 is involved in regulation of* in vitro *oxidative stress resistance and survival in human macrophages.*

**Figure 5.**
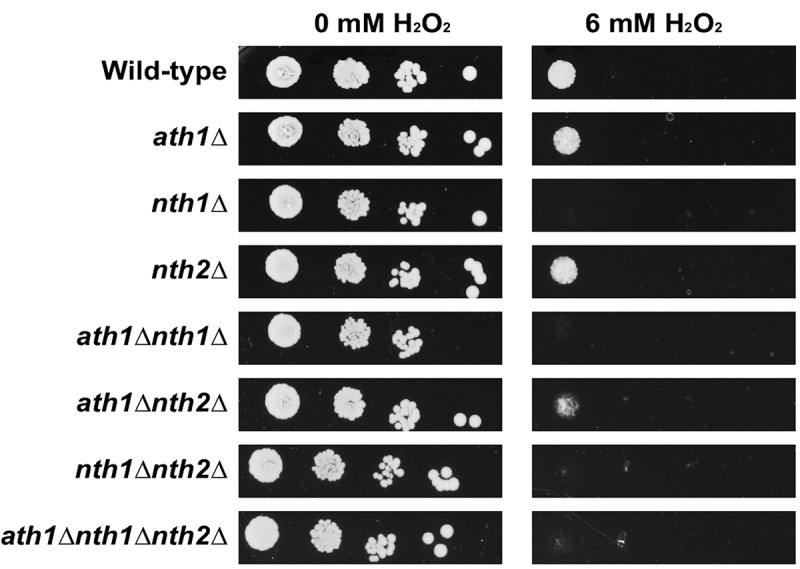
**Oxidative stress resistance of different trehalase mutant strains**. The different strains were grown overnight in SC 100 mM glucose. After 3 washing steps, cells were spotted on YNB plus 100 mM glucose agar plates containing 6 mM of H_2_O_2_ and grown at 37°C for 72 hours

Inside the human body, fungal pathogens are continuously exposed to different types of environmental stresses [74]. As trehalose is a disaccharide important for stress resistance, we assessed the growth phenotype of the trehalase deletion strains upon stress treatment. Therefore, cells were grown in the presence of different relevant stresses: oxidative stress (H_2_O_2_), salt stress (NaCl) and cell wall stress (Calcofluor White, CFW). Deletion of *ATH1* and/or *NTH2* did not affect the growth in the presence of these stressors, while strains lacking *NTH1* were more sensitive toward oxidative stress (Figure 5, Fig S5 and Fig S6). This phenotype is observed in all strains lacking *NTH1* with no additive effect of the other trehalases (Fig S6). Furthermore, all *nth1∆* strains showed a decreased survival after acute exposure to different concentrations of hydrogen peroxide (Fig S7). The disruption of *NTH1* did not affect growth in the presence of salt nor cell wall stress (Fig S5).

**Figure 6.**
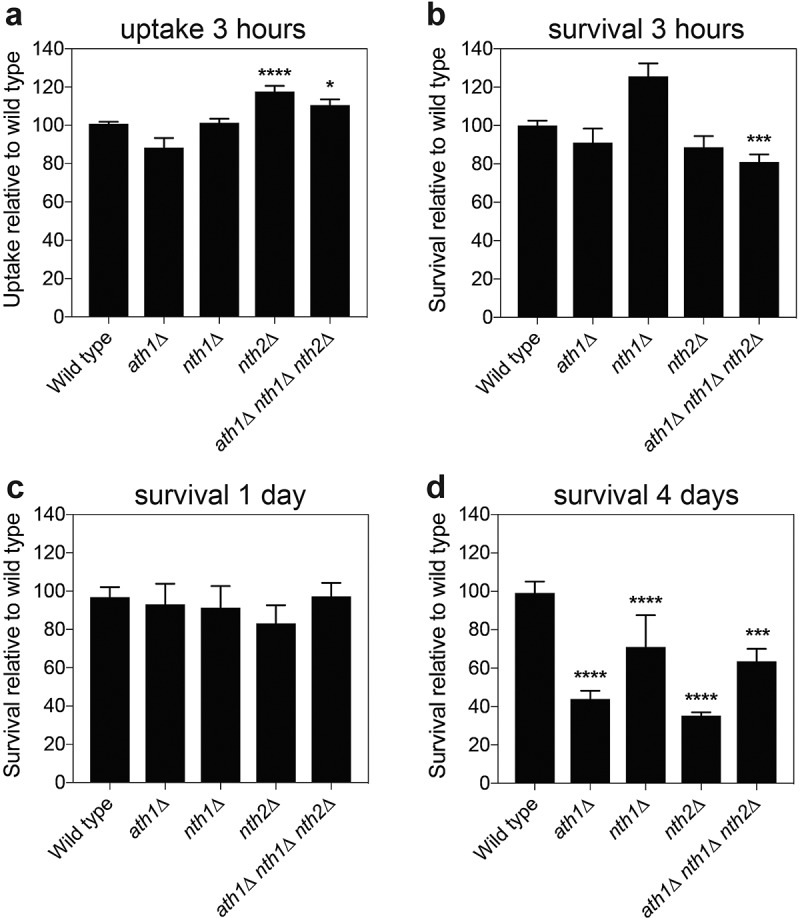
***Candida glabrata* trehalase survival inside macrophages**. Differentiated human macrophages were infected with the indicated *C. glabrata* strain at multiplicity of infection (MOI) 1:1, after which the cells were plated for CFU counting at the indicated days (b-d). Additionally, at 3 hours post infection, also the uptake of each strain was counted (a). Average survival relative to the wild type strain is shown with SEM of minimal three experiments, statistical Kruskal-Wallis test was performed with Dunn’s correction (*, P ≤ 0,05; **, P ≤ 0,01; *** P ≤ 0,001 and ****, P ≤ 0,0001), comparing the survival of all strains to wild type

One of *C. glabrata*’s virulence traits is its ability to survive and replicate inside human macrophages [75–78]. This survival demands for a high stress resistance as the yeast cells encounter a change in pH, nutrient limitation and oxidative stress within the macrophages [79,80]. As a difference in oxidative stress resistance was observed for the *nth1∆* strains, their survival in macrophages was investigated. In addition, the lab of prof. Hube at the Hans Knöll Institute, Jena, Germany investigated the transcriptional response of *C. glabrata* cells to incubation with a macrophage like-murine RAW cell line. The transcriptome showed that in comparison to growth in complex medium, the expression level of the acid trehalase gene *ATH1* was overall very high, slightly dropped during the first hour and went up again (Fig S8A). Furthermore, the expression of *NTH1* and *NTH2* increased within the first hour of incubation, with the transcript level especially of *NTH2* remaining very high at all later timepoints inside the macrophage-like cells (Fig S8A). We therefore also tested if the deletion of the trehalase enzymes could influence the survival in human macrophages, especially at later timepoints, when trehalose utilization may play an important role. To this end, we infected human monocyte-derived macrophages with the different *C. glabrata* strains at a multiplicity of infection (MOI) of 1:1 for a long-term co-incubation. At different timepoints (3 hours, 1 day and 4 days), the macrophages were lysed and the *C. glabrata* cells were used for transcript determination by microarrays and plated for CFU counting to determine survival (Fig S8B and 6A-D). To ensure the difference in survival was due to killing by macrophages and not to altered uptake or escape and extracellular growth of the *Candida* cells, we measured the uptake rate and ensured that our samples did not contain extracellular *C. glabrata*. The uptake by the immune cells was nearly identical for all strains, with a somewhat increased uptake of *nth2∆* (Figure 6a). After 3 hours, more *nth1∆* than wild type yeasts were re-isolated (not statistically significant), while the *nth2∆* and the triple deletion mutant were found to be slightly reduced in surviving numbers. These small differences were gone after one day of incubation, with the strains adapting to long-term survival (Figure 6c). After 4 days of incubation, all single deletion strains and the triple deletion strain showed a significantly decreased survival (down to 44% for *ath1∆*, 65% for *nth1∆*, 35% for *nth2∆* and 64% for *aht1∆ nth1∆ nth2∆*) compared to the wild type strain (Figure 6d). The requirement for these genes in human macrophages was reflected by the continuously increased transcript levels as determined in parallel by microarray experiments (Fig S8B).

### *Trehalase plays only a minor role in the virulence of* C. glabrata *in a mouse systemic infection model*

**Figure 7.**
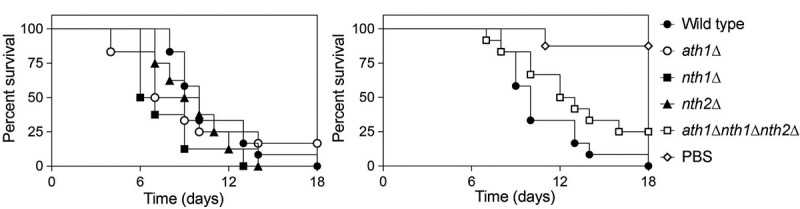
**Mice survival after systemic infection**. Mice were immunosuppressed with 75 mg/kg dexamethasone and received 5 . 10^7^ *C. glabrata* cells via injection in the lateral tail vain. Wild type (●), *ath1*∆ (○), *nth1*∆ (■), *nth2*∆ (▲) and *ath1∆nth1∆nth2∆* (Δ) or PBS as a control (□) were tested. The mice were monitored two times per day for 18 days. When they reached humane endpoints, they were sacrificed. Statistical analysis by the use of a log-rank test was performed comparing the deletion strains to the wild type (*, P ≤ 0,05; **, P ≤ 0,01; *** P ≤ 0,001 and ****, P ≤ 0,0001)

In order to investigate whether the trehalase enzymes are involved in *C. glabrata* virulence in a systemic infection, mice were infected intravenously with the different deletion strains. Experimental infection of immunocompetent mice with *C. glabrata* generally does not cause mortality [81]. We confirmed this in a pilot experiment during which we determined the optimal concentration of the immunosuppressant dexamethasone (Fig S9). 75 mg/kg dexamethasone was chosen to be given to the mice at day −3, 0, 7 and 14. For each deletion strain, at least 8 mice were included in the experiment (Figure 7). The mice infected with the *nth1*∆ strain appeared ill more rapidly and died earlier compared to the wild type (p-value of 0.0184 in log-rank test). The mice infected with the triple deletion strain showed no significant difference in survival compared to mice infected with the wild type strain (p-value 0.0873 in log-rank test). Based on the twice per day monitoring, it was clear that mice infected with the triple deletion mutant did not show disease phenotypes such as ruffled hair, similar to the PBS control mice and different from mice infected with the wild type strain. It should be noted that two mice of the PBS control group died, which is most probably due to the immunosuppression of the mice and the duration of the experiment. The other mice of the PBS control group showed a healthy appearance throughout the experiment.

## Discussion

This work focused on the characterization of the different *C. glabrata* trehalase enzymes and their role in virulence. To our knowledge, there are no prior publications in which a mutant lacking all three trehalase enzymes in a pathogenic yeast strain is characterized. Comparison with the *S. cerevisiae* trehalase enzymes resulted in the identification of three trehalase enzymes in *C. glabrata*: Ath1, Nth1 and Nth2. When comparing the *C. glabrata* trehalase enzymes to those present in other pathogenic and nonpathogenic fungi two clusters can be distinguished: a cluster of neutral trehalase enzymes and a cluster of acid trehalase enzymes. Both types of these proteins have a different structure: the neutral trehalases contain one large domain for trehalase activity containing the active site and binding site for the substrate, next to a small calcium binding domain. The acid trehalase contains one predicted transmembrane domain and two domains for trehalase activity [82].

Single and multiple deletions of the encoding genes were constructed and functionally characterized. We showed that Ath1 is important for the rapid hydrolysis of extracellular trehalose, a phenotype on which the rapid identification of *C. glabrata* in the hospital is based [42–48]. In different organisms, the acid trehalase enzyme is present on the outside of the cells in order to hydrolyze exogenous trehalose [37,39,40,51,68,69]. Our microscopy data, where Ath1 was tagged to a fluorophore, showed that Ath1 is present both on the outside of the cells as well as in the vacuole. Furthermore, high acid trehalase activity was measured in complete cells, which was lost upon preparation of protoplasts. At the same time, the fluorescent signal of the labeled Ath1 at the outside of the cells is lost. This shows that Ath1 is localized in the periplasmic space, similar to the situation in *S. cerevisiae* [37]. We hypothesize that this localization helps the cells to quickly hydrolyze extracellular trehalose to glucose, which is then taken up by the glucose transporters in the plasma membrane [83]. Zilli et al. showed that Ath1 is also secreted to the outside of the cells and is released into the medium [41]. As the activity of Ath1 is very high, we hypothesized that *C. glabrata* cells would have a competitive advantage over other microbes in those conditions where there is trehalose present, such as in the gut. In humans, *Candida* is present in the gastrointestinal tract as part of the normal microbiota [84]; hence, we hypothesized that Ath1 might play a relevant role in the colonization of *C. glabrata* at this body site. This appeared not to be the case, as in our experiments, the *ath1∆* strain showed a wild type phenotype in the mouse model of GI colonization. Interestingly, additional deletion of *NTH1* and *NTH2* resulted in a clear drop in GI colonization. This indicates that trehalase activity is indeed important to achieve persistent colonization, however, any of the enzymes could contribute to this.

In *C. albicans* and in *C. parapsilosis*, the acid trehalase Atc1 is a stress-responsive protein and is important for *in vitro* stress resistance. Moreover, mice infected intravenously with *C. albicans* or *C. parapsilosis* cells lacking their acid trehalase enzyme show an increased survival, suggesting a role for *ATC1* in virulence [49,51,85]. None of these phenotypes are observed for the acid trehalase of *C. glabrata*. However, after 4 days of infection in macrophages, Ath1 seemed to play a role in the survival of *C. glabrata* cells. We hypothesize that through binding to trehalose as a stress protectant, many proteins are cycled to the vacuole for autophagy [77]. As such, it is likely that Ath1 is present in the vacuole which allows trehalose hydrolysis in the vacuole, as is the case in *S. cerevisiae* [37], providing energy to the cells. These data confirm that the function of Ath1 is different from its orthologous enzymes in other *Candida* species, possibly explaining why the specific trehalose test is used to diagnose *C. glabrata*.

Similar to *S. cerevisiae, C. glabrata* Nth1 is important for stress tolerance, specifically toward oxidative stress, which is encountered inside immune cells. Deletion of *NTH1*, as well as the other trehalases, indeed results in a lower survival upon engulfment by macrophages. We hypothesize that the reason for this is the lower tolerance toward oxidative stress for this deletion strain. The small enhanced virulence of the *nth1∆* strain in the systemic mouse model was unexpected. However, other examples are known, where a mutant strain shows opposite phenotypes in different virulence experiments [86,87]. Important to mention is the fact that the mice in the experiment are immunocompromised, leaving out a role for the immune system, in which the trehalase deletion strains would survive less well. The role of Nth2 remains unclear. Under *in vitro* conditions, the *NTH2* gene is rapidly upregulated inside macrophages and its deletion results in a lower survival under these conditions. Its higher expression in macrophages resembles the higher *ScNTH2* expression during the onset of stationary phase growth [88]. This suggests that the cells encounter conditions in the macrophages similar to the ones they encounter when reaching stationary phase. The decreased survival of the mutant strain after 4 days remains to be investigated.

The *ath1∆ nth1∆ nth2∆* strain did not show detectable trehalase activity in any growth phases, indicating that these three genes are all the trehalase enzymes present in *C. glabrata*. In general, the triple deletion strain showed results consistent with the single deletion strains. The triple deletion strain showed a decreased survival on hydrogen peroxide due to the absence of *NTH1*. This strain is also impaired in survival in human macrophages after 4 days of inoculation. Additionally, the triple deletion strain was unable to use trehalose as a carbon source, as is also the case for the *ATH1* deletion strains.

Based on our animal experiments, we can conclude that none of the single enzymes alone constitutes an interesting antifungal drug target, however a combined inactivation of these enzymes is of interest as the triple deletion showed a decreased survival in human macrophages and was fully cleared from the murine GI tract. Additionally, the mice infected intravenously with the triple deletion strain appeared more fit (based on our monitoring of their behavior) compared to the wild type control. As they share trehalose as a substrate, variants of trehalose may cause inhibition of all three enzymes. As far as the single targets are concerned, inactivation of Ath1 may already be interesting, as this enzyme is required for cells to grow on trehalose and its deletion results in a decrease in survival inside macrophages.

*C. glabrata* infections are frequent in immunocompromised patients, where the endogenous *C. glabrata* can disseminate from the GI tract to cause invasive infection [89]. These results seem promising and therefore, we propose that a drug targeting all three trehalases could have a positive effect on the outcome of a *C. glabrata* infection. As all enzymes have the same substrate, the search for a competitive inhibitor seems the most straight-forward. Structural analogues of trehalose can act as a competitive inhibitor of trehalase [90]. Validamycin A, a well-known trehalase inhibitor, is already used in food crops to prevent fungal infections [91]. This compound showed to have weak antifungal activity against *C. albicans* whereas a strong effect was observed against *A. flavus* [92,93]. Based on this work, we propose to test different structural analogues of trehalose for their antifungal activity against *C. glabrata*.

Even though humans cannot synthesize trehalose, their genome encodes for one trehalase. The human trehalase enzyme can be found in the intestines and kidneys but also in the urinary tract, where it is used as a marker for renal tubular damage [94,95].The human enzyme clusters together with the fungal neutral trehalases (Figure 1). It shows only 27% amino acid identity to both Nth1 and Nth2 of *C. glabrata*, but the amino acids involved in the substrate binding and the active site are conserved (supplementary data). However, a deficiency of the human trehalase is rare and causes diarrhea due to an intolerance to trehalose enriched food, such as mushrooms [96]. This should be taken into account when looking for competitive inhibitors of the *C. glabrata* trehalase enzymes.

## Supplementary Material

Supplemental Material
