# Supplementary material for: The involvement of the Candida glabrata trehalase enzymes in stress resistance and gut colonization
Source: Virulence. 2020 Dec 28;12(1):329–45. doi: 10.1080/21505594.2020.1868825 (PMC7808424; doi:10.1080/21505594.2020.1868825)
Supplement: Supplemental Material [file KVIR_A_1868825_SM6007.zip › SUPPLEMENT/Supplementary data - alignment neutral trehalases and human.pdf]

CLUSTAL O(1.2.4) multiple sequence alignment

AA of active site - AA conferring **substrate binding**

```
Human_TREH          ------------------------------------------------------------ 0
CgNTH2              ----MPTLTFY--------------------------------LGADDAVDGGVKSKSMA 24
ScNTH2              MVDFLPKVTEINPPSEGNDGEDNIKPLSSGSEQRPL----KEEGQQGGRRHHRRLSSMHE 56
CgNTH1              ---------MSDPMDEGP--------LGKGAVATPAMVE-KVANESPKKTRPRRLSSLNE 42
ScNTH1              ------------------------------------MSQVNTSQGPVAQGRQRRLSSLSE 24
S_Pombe_Ntp1        -------------------------------------MPSKFSSKYVD--TE---AISND 18
A_nidulans_TreB     -------------------------------MDDSA-LPSNTSNGINGRTAH---RRSSS 25

Human_TREH          ------------------------------------------------------------ 0
CgNTH2              FSDPFHKNVKVQSPEPEPVDA--KEKKKLNRTRTMSVFYNVADFKD-DEEIRKHKLKRRA 81
ScNTH2              YFDPFSNAEVYYGPITDPRKQSK--IHRLNRTRTMSVFNKVSDFK---NGMKDYTLKRRG 111
CgNTH1              FYDPFSTAEVYYGPATDPRKQKKS-STKLNRTRTMSVFDNTSIAKT-TASFGQFQLKRRG 100
ScNTH1              FNDPFSNAEVYYGPPTDPRKQKQAKPAKINRTRTMSVFDNVSPFK--KTGFGKLQQTRRG 82
S_Pombe_Ntp1        DDNPFATAKSYYSKDTDLSTR-----VSAGRPRTLSTSMEASAAPTIPE---LKNLRRRG 70
A_nidulans_TreB     GGDPFQHPDIYYGNPESVERI-------KNRRRAFSSSLKSFNRQDFHEMLGDRNT-RRG 77

Human_TREH          ----------------MPGRTWELCL-------------------LLLLGLGLGSQEALP 25
CgNTH2              SEDDTYISSKGTRRLFVKDVDWTLKELLQNEDTDQNFQITVEDTGPKVIKLGTANSAGYN 141
ScNTH2              SEDDSFLSSQGNRRFYIDNVDLALDELLASEDTDKNHQITIEDTGPKVIKVGTANSNGFK 171
CgNTH1              SEDDSLTASQGNRKFFIEDVDGTLEELLSSEDTDRNYQITIEDTGPKVLKVGTANSGGYK 160
ScNTH1              SEDDTYSSSQGNRRFFIEDVDKTLNELLAAEDTDKNYQITIEDTGPKVLKVGTANSYGYK 142
S_Pombe_Ntp1        SL-D----EHKQPRKFLVDVDKTLNALLESEDTDRNMQITIEDTGPKVVSLGSASSGGYR 125
```

```
A_nidulans_TreB      SMDP----TSGNPRKFLIDVDATLHSLLEREDSDRNMQITIEDVGPKVFSLGTAASHGYN  133
                                     : .    *                       :. :* . . .

Human_TREH           PPCESEIYCHGELLNQVQMAKLYQDDKQFVDM------PLSIAPEQVLQ-----------  68
CgNTH2               HIDIRGTYMLSNLLQELTIAHNFGQRQLYLPESRLNENPVHRLKRVIETHFWDGLTRRLD  201
ScNTH2               HVNVRGTYMLSNLLQELTIAKSFGRHQIFLDEARINENPVDRLSRLITTQFWTSLTRRVD  231
CgNTH1               HVNIRGTYMLSNLLQELTIAKSFGRHSIFLDEARINENPVNRLSRLITNQFWNNLTRRID  220
ScNTH1               HINIRGTYMLSNLLQELTIAKSFGRHQIFLDEARINENPVNRLSRLINTQFWNSLTRRVD  202
S_Pombe_Ntp1         LYELRGTYQLSNLLQELTLAKDYGRRYILLDERRLNENPVNRLSRLIKGTFWDALTRRID  185
A_nidulans_TreB      RFDVRGTYMLSNLLQELTIAKDYGRKQIVLDEERLSENPVSRLSRLIKNSFWNSLTRRID  193
                            *  .:**::: :*: :      :        *:    . :

Human_TREH           --TFTELSRDHNHSIPREQLQAFVHEHFQAKGQELQPWTPADWKDSPQFLQKISDAK---  123
CgNTH2               LNTIGEAAQEKKIDTPEAK--------------NPRVYVPYDCPEMYEYFIQASQMNPSV  247
ScNTH2               LYNIAEIARDSKIDTPGAK--------------NPRIYVPYNCPEQYEFYIQASQMNPSL  277
CgNTH1               LNNIGEIASDTKVDTPEAK--------------NPRVYVPYNCPEQYEFYIQASQMNPSM  266
ScNTH1               LNNVGEIAKDTKIDTPGAK--------------NPRIYVPYDCPEQYEFYVQASQMHPSL  248
S_Pombe_Ntp1         ASVLDVICRDTK-DRSGSH--------------VNRIYVPKAEQEMYEYYVRAAKERPYL  230
A_nidulans_TreB      GRNIEVAGRDPK-DWTDDP--------------RPRIYVPPGAPEQLEYYRRIAEEKPEL  238
                        .     : : .                     : :.*    :  ::  : :. .

Human_TREH           -----------LRAWAGQLHQLWKKLGKKMKPEVLSHPERFSLIYSEHPFIVPGGRFVEF  172
CgNTH2               KLEVEYLPKDITPEYVQSLNETPGLLAIAMDKH--VNPTTGETSLVGFPYVFPGGRFNEL  305
ScNTH2               KLEVEYLPKDITAEYVKSLNDTPGLLALAMEEH--VNPSTGERSLVGYPYAVPGGRFNEL  335
CgNTH1               KLEVEYLPKDITPEYVKSLNATPGLLALAMEEH--VNPATGEVSLIGYPYAVPGGRFNEL  324
ScNTH1               KLEVEYLPKKITAEYVKSVNDTPGLLALAMEEH--FNPSTGEKTLIGYPYAVPGGRFNEL  306
S_Pombe_Ntp1         NLQVEYLPEEITPEWVRDVNDKPGLLALAME-K--YQDDEGNTHLRGVPFVVPGGRFNEL  287
A_nidulans_TreB      RLDVQELAAEITPEYVRDLNEKPGLLALAMEEK--YDEKTGKTDFAGVPFVVPGGRFNEL  296
```

```
                                  :. .::     *.  *. .   .    .      *: .***** *:

Human_TREH                YYWDSYWVMEGLLLSEMAETVKGMLQNFLDLVKTYGHVPNGGRVYYLQRSQPPLLTLMMD  232
CgNTH2                    YGWDSYLITLGLLASKRTDLARGMVEHFIFEIEHYGKILNANRTYYLCRSQPPFLTDMAN  365
ScNTH2                    YGWDSYLMALGLIESNKVDVARGMVEHFIFEIDHYSKILNANRSYYLCRSQPPFLTDMAL  395
CgNTH1                    YGWDSYMMALGLLESNKVDVARGMVEHFIFEIEHYGKILNANRSYYLCRSQPPFLTDMAL  384
ScNTH1                    YGWDSYMMALGLLEANKTDVARGMVEHFIFEINHYGKILNANRSYYLCRSQPPFLTEMAL  366
S_Pombe_Ntp1              YGWDSYFESLGLLVDDRVDLAKGMVENFIFEITYYGKILNANRTYYLLRSQPPFLTDMAL  347
A_nidulans_TreB           YGWDSYMESLGLLASNRVDLAKAMVINFCFCIKHYGKILNANRSYYLTRSQPPFLTDMAL  356
                          * ****    **:  . .: .:.*: :*   :  *.:: *..* *** *****:** *

Human_TREH                CYLTHTN------DTAFLQENIETLA-LELDFWTKNRTVSVSLEGKNYLLNRYYVPYGGP  285
CgNTH2                    VTFNGMGGNKNPIAVDLLRRAFRAAIKEYKTVWMAKPRLDEK-----TGLSCYHPDGVGI  420
ScNTH2                    LVFEKIGGKNNPNAIQLLKRAFRAAIKEYKEVWMSSPRLDSL-----TGLSCYHSDGIGI  450
CgNTH1                    VVFNRIGGDNNPNAIDFLKRSFKAAIKEYKTVWMAHPRLDPE-----TGLSCYHPDGLGI  439
ScNTH1                    VVFKKLGGRSNPDAVDLLKRAFQASIKEYKTVWTASPRLDPE-----TGLSRYHPNGLGI  421
S_Pombe_Ntp1              RVYERIK--NEEGSLDFLHRAFSATIKEYHTVWTATPRLDPK-----TGLSRYRPGGLGI  400
A_nidulans_TreB           RVYDRIQ--NEPGAMDFLRHAILAAIKEYYSVWMAEPRLDPV-----SGLSRYRSPGIGV  409
                                          :*:. : :       .*     :.         *. *     *

Human_TREH                RPESYSKDVE-------------L--------ADTLPEGDREALWAELKAGAESGWDFSS  324
CgNTH2                    PPETEPEHFDTKLRPYAKKYNVTIPEFRRMYNAKEVHEPELDEFFLHDRGVRESGHDTTY  480
ScNTH2                    PPETEPDHFDTILLPYAEKYNVTLEKLRYLYNEGMIKEPKLDAFFLHDRAVRESGHDTTY  510
CgNTH1                    PPETEPTHFDSILTPYAAKYYCTIPEFIAMYNDGSVKEPHLDEFFLHDRGVRESGHDTTY  499
ScNTH1                    PPETESDHFDTVLLPYASKHGVTLDEFKQLYNDGKIKEPKLDEFFLHDRGVRESGHDTTY  481
S_Pombe_Ntp1              PPETEASHFEHLLRPYMEKYHMTLEEFTHAYNYQQIHEPALDEYFVHDRAVRESGHDTTY  460
A_nidulans_TreB           PPETEASHFLHLLTPYAEKHGMEFKEFVQAYNYGKVKEPELDEYFMHDRAVRESGHDTSY  469
                           **:   ..              :           : *   :  : . :.  *** * :
```

```
Human_TREH           RWLIGGPNPNSLSGIRTSKLVPVDLNAFLCQAEELMSNFYSRLGNDS-------------  371
CgNTH2               RLE----------G-VCAYLATIDLNSLLYKYEVDIANFIKEFCNDKYVDPYD-------  522
ScNTH2               RFE----------G-VCAYLATIDLNSLLYKYEKDIAFVIKEYFGNEYKDEND-------  552
CgNTH1               RFE----------G-VCAYLATIDLNSLLYKYEVDIANFIEKYHNDEYHDPFD-------  541
ScNTH1               RFE----------G-VCAYLATIDLNSLLYKYEIDIADFIKEFCDDKYEDPLD-------  523
S_Pombe_Ntp1         RLE----------K-VCADLATVDLNSLLYKYETDISHVILEYFDDKFVLPNG-------  502
A_nidulans_TreB      RLE----------R-VCGNLATVDLNSLLYKYEVDIARVIRVYFKDKLEIPVEFRTPATK  518
                     *                . *. :***::* : *  :: .      :.

Human_TREH           -----QATKYRILRSQRLAALNTVLWDEQTGAWFDYDLEKKKKNREFYPSNLTPLWAGCF  426
CgNTH2               -KSVTTSEDWMNLANKRKERIKFYMWDEEAGFFFDYNVKTEKRTSYESATTFWAMWAGVA  581
ScNTH2               -GTVTDSEHWEELAELRKTRINKYMWDEDSGFFFYYNTKLKCRTSYESATTFWSLWAGLA  611
CgNTH1               -GTVTNSAYWRDLAAKRKENITKYMWDEETGFFYDYNIKVKCRTNYESATTFWSLWAGLA  600
ScNTH1               -HSITTSAMWKEMAKIRQEKITKYMWDDESGFFFDYNTKIKHRTSYESATTFWALWAGLA  582
S_Pombe_Ntp1         --TIETSAIWDRRARARRAAMEKYLWSEADSMWYDYNTKLETKSTYESATAFWALWAGVA  560
A_nidulans_TreB      DIQSESSSVWDRRARRRKMRMDTYLWDEEKGMYFDYDTVKQERTNYESATTLWAMWAGLV  578
                           :  :      *   :   :*.:  . :: *:   : :.     : :  :***

Human_TREH           SDPGVA---DKALKYLED---NRILTYQYGIPTSLQKTGQQWDFPNAWAPLQDLVIRGLA  480
CgNTH2               SPQEAEIMVKKAVPKLETLGGIVACTESSRGPIAIDRPYRQWDYPFGWAPHQILAWKGFS  641
ScNTH2               TEEQAKITVEKALPQLEMLGGLVACTEKSRGPISIDRPIRQWDYPFGWAPHQILAWKGLS  671
CgNTH1               TPEQAKKMVELALPKLEMLGGLVACTEESRGPISIDRPHRQWDYPFGWAPHQILAWEGLN  660
ScNTH1               TKEQAQKMVEKALPKLEMLGGLAACTERSRGPISISRPIRQWDYPFGWAPHQILAWEGLR  642
S_Pombe_Ntp1         TPRQAAKFVDVSLPKFEVAGGIVAGTKRSLGKVGLDNPSRQWDYPNGWSPQQILAWYGLI  620
A_nidulans_TreB      TPRQASAMITKALPRFEEFGGIVSGTEESRGAVGLNRPTRQWDYPYGWAPQQMLAWTGFA  638
                     :   .      ::  :*        *       .:..  :***:* .*:* * *.  *:
```

```
Human_TREH           KAPLRRAQEVAFQLAQNWIRTNFDVYS-QKSAMYEKYDVSNGGQPGG-----------GGE  529
CgNTH2               NYGY---DNIARRLVYRWLYMMTKAFVDYNGIVVEKYDVTKVTDPHKITAEYGNQGVDFK  698
ScNTH2               AYGY---QQVATRLAYRWLYMITKSFVDYNGMVVEKYDVTRGTDPHRVDAEYGNQGADFK  728
CgNTH1               DYGY---TSVATRLAYRWLFMMTKAFVDYNGIVVEKYDVTRGTDPHRVDAEYGNQGADFK  717
ScNTH1               SYGY---LTVTNRLAYRWLFMMTKAFVDYNGIVVEKYDVTRGTDPHRVEAEYGNQGADFK  699
S_Pombe_Ntp1         RYGY---EEETRRLVYRWLYTITKSFVDFNGIVVEKYDLTRPVDPHRVEAEYGNQGVNIK  677
A_nidulans_TreB      RYGY---QEEAERLAYKWLYMITKAFVDFNGVVVEKYDVTRPIDPHRVDAEYGNQGVDFK  695
                               : :*. .*:    . :   :. : ****::.  :*            . :

Human_TREH           YEVQEGFGWTNGVVLMLLDRYGDRLTSGAKLAFLEPHCLAATLLPSLLLSLLPW*-----  583
CgNTH2               GYAREGFGWVNSSFLLGLQYLDNHAIR-----------ALGACIPPKPFLNGLRAKEKARY  748
ScNTH2               GVATEGFGWVNTSYLLGLKYMNNHARR-----------ALAACSPPLPFFNSLKPSEKKLY  778
CgNTH1               GVAKEGFGWVNTSYILGLKFMNSHARR-----------ALGACIPPAPFFNSLKDDEKSIY  767
ScNTH1               GAATEGFGWVNASYILGLKYMNSHARR-----------ALGACIPPISFFSSLRPQERNLY  749
S_Pombe_Ntp1         GVAREGFGWVNASYEVGLTFCNSHMRR-----------ALGACTTPDVFFAGIKEESLPA-  726
A_nidulans_TreB      GAPREGFGWVNASYVYGLEMLNAHQRR-----------ALGAVTPWETYSKAVSAQGSDTV  745
                         *****.*      *   . :             .*.*          :

Human_TREH           ---------- 583
CgNTH2               GL*------- 750
ScNTH2               YL*------- 780
CgNTH1               GL*------- 769
ScNTH1               GL*------- 751
S_Pombe_Ntp1         FENLSIHKN* 735
A_nidulans_TreB      LENRSE*--- 751
```
