# Supplementary material for: The involvement of the Candida glabrata trehalase enzymes in stress resistance and gut colonization
Source: Virulence. 2020 Dec 28;12(1):329–45. doi: 10.1080/21505594.2020.1868825 (PMC7808424; doi:10.1080/21505594.2020.1868825)
Supplement: Supplemental Material [file KVIR_A_1868825_SM6007.zip › SUPPLEMENT/Supplementary Table 1.docx]

**Supplementary Table 1 – Strains, plasmids and primers used in this study**

List of strains:

| **Species** | **Strain** | **Genotype** | **Source** |
| --- | --- | --- | --- |
| *C. glabrata* | 311 | *his3∆:::FRT trp1∆:::FRT leu∆:::FRT* | [1] |
| *C. glabrata* | *Cg*BM1 | *ath1*∆:::FRT | This work |
| *C. glabrata* | *Cg*BM2 | *nth1*∆:::FRT | This work |
| *C. glabrata* | *Cg*BM3 | *nth2*∆:::FRT | This work |
| *C. glabrata* | *Cg*BM4 | *ath1*∆:::FRT *nth1*∆:::FRT *NTH2* | This work |
| *C. glabrata* | *Cg*BM5 | *ath1*∆:::FRT *NTH1* *nth2*∆:::FRT | This work |
| *C. glabrata* | *Cg*BM6 | *ATH1 nth1*∆:::FRT *nth2*∆:::FRT | This work |
| *C. glabrata* | *Cg*BM7 | *ath1*∆:::FRT *nth1*∆:::FRT *nth2*∆:::FRT | This work |

List of plasmids:

| **Plasmid** | **Description** | **Used for** | **Source** |
| --- | --- | --- | --- |
| pYC44 | NatMX cassette flanked by FRT sites | Construction of deletion cassette | [2] |
| pYC48 | HA tag, NatMX cassette flanked by FRT sites | Construction of reintegrant | [2] |
| pYC56 | mCherry, NatMX cassette flanked by FRT sites | Construction of mCherry tagged fusions | [2] |
| pLS9 | Flippase NatMX | Removal of resistance marker between FRT sites | Kindly provided by Alejandro De Las Peñas & Irene Castaño |
| pLS10 | Flippase hph | Removal of resistance marker between FRT sites | This work |
| p58 | PYC-gRNA-uni-hph | Amplification hygromycin marker | [3] |
| pBM13 | pYC56-*ATH1* | Construction endogenously tag trehalase-mCherry | This work |
| pBM14 | pYC56-*NTH1* | Construction endogenously tag trehalase-mCherry | This work |
| pBM15 | pYC56-*NTH2* | Construction endogenously tag trehalase-mCherry | This work |

List of primers:

| **Primer** | **Name** | **Sequence** | **used for** |
| --- | --- | --- | --- |
| 7883 | pLS10-*HPH*-Fw | GACAACGTATACTCATCAGATAACAGCAATA | Checking pLS10 plasmid |
| 9064 | NatMx_Rv | cgtcaagactgtcaaggaggg | checking insertion pYC vectors |
| 9065 | NatMX_Fw | catcatctgcccagatgcgaag | checking insertion pYC vectors |
| A2047 | vector background Rv | cagctatgaccatgattacg | Checking insertion terminator fragment in pYC56 |
| A9050 | mCherry Rv | CTTCACCTTGTAGATGAAC | Checking insertion ORF fragment in pYC56 |
| B1222 | NatMX Fw | cgattcgatactaacgcc | Checking insertion terminator fragment in pYC56 |
| B2011 | *NTH2* control Rv | CGTGTCTAGTATACAATGCTC | Checking transformants n*th2∆* |
| B3220 | pLS10-*HPH*-Rev | GTAATACAGTCAAATTGCAGTACTCTGC | Checking pLS10 plasmid |
| B4165 | pYC backbone Fw | GTAAAACGACGGCCAGTG | checking insertion pYC vectors |
| C2950 | vector background Fw | taatacgactcactataggg | Checking insertion ORF fragment in pYC56 |
| C3161 | *NTH2* control Fw | CAACGGCAACAACTTCC | Checking transformants n*th2∆* |
| C3162 | *NTH2* control Rv | TTAGCCTTGATTCTGGC | Checking transformants n*th2∆* |
| C3177 | *ATH1* control Fw | ACGGATGATTCAAAGGGG | Checking transformants *ath1∆* |
| C3178 | *ATH1* control Rv | CTTGAGTAAGTGTTCTCACC | Checking transformants *ath1∆* |
| C3183 | *NTH1* control Fw | CACGAGCTATAGTCCACAG | Checking transformants n*th1∆* |
| C3184 | *NTH1* control Rv | GGAAGATGGAGTGTCTACC | Checking transformants n*th1∆* |
| C3835 | *NTH1* control Rv | TGGCCATGACTACCGAAGTC | Checking transformants n*th1∆* |
| C6240 | *HPH*-Fw-NotI-XhoI | GATCGATGCGGCCGCCTCGAGcacAGATCTGTTTAGCTTGC | Construction pLS10 plasmid |
| C6241 | *HPH*-Rv-NotI | GATCGATGCGGCCGCtCAAaggttaccccagttgg | Construction pLS10 plasmid |
| C6315 | *ATH1*-Fw-del-FRT | CTAAATTTCTGATTATAACACAACACAACACACACACACAAACTCTGTAAATAATAACAAGACAACACTGCAAGTAGCGAGGTCTGGAAAGAGAAGTACAgctctagaactagtggatcc | Construction deletion cassette *ATH1* |
| C6316 | *ATH1*-Rv-del-FRT | AATATTGTAAGTAATTTCAGATTTTTTTATCCAGATTATAAAAAATAAACTAATTACTCTGCCAAGTAATTATGTGTAGATATGAGCTAGATCCCCAGTTggaacaaaagctgggtacc | Construction deletion cassette *ATH1* |
| C6317 | *NTH1*-Fw-del-FRT | ATCAACTCCCATAAATTACTAGTGAAAACCCTCTCGCTGAAGGAAAACTAAATCTAAATCTAAATCTAAATCCAAAACTCACCAAAAAAATAGTGCAATAgctctagaactagtggatcc | Construction deletion cassette *NTH1* |
| C6318 | *NTH1*-Rv-del-FRT | GATCTTTTCTCAATAGAATCATAAAATTATTCTAGACAGCATTGTGAATATTTTAAATACACTACATATCATCTGGAATATAAAGATAAAGAGTCTCAATggaacaaaagctgggtacc | Construction deletion cassette *NTH1* |
| C6319 | *NTH2*-Fw-del-FRT | CAGGCAAAACTTGAAAATAATAACCGAAACTAAATTAAATAATTTAACTTACATCAAAATAACATAATAACAAAAGTACACATTACACATTACACTCGCGgctctagaactagtggatcc | Construction deletion cassette *NTH2* |
| C6320 | *NTH2*-Rv-del-FRT | AAATCGAACAGTCCGTAGAGTCATCTTTAAATATGTAATTGACGTGCCAAAAATTTCAAATGCCTTACGAGATGATTAATATCTAAAAACGTCGAGCAATggaacaaaagctgggtacc | Construction deletion cassette *NTH2* |
| C8497 | *ATH1* control Rv | GTCCTGTGTTCGATCCAC | Checking transformants *ath1∆* |
| C9381 | ORF ATH1 | GGTGGAGATAAACCGATC | checking insertion mCherry tag genome |
| C9385 | ORF NTH1 | CCATTGATCTCAACTCAC | checking insertion mCherry tag genome |
| C9388 | ORF NTH2 | CTTCATGACCGAGGTGTG | checking insertion mCherry tag genome |
| D1728 | CgATH1-mCherry_Rv | GCCCTTGCTCACCATTAGGATCCCTTCTTGAAGGACAATTTCCTTGATGG | Construction pBM13 |
| D84 | CgATH1-ORF_Fw_EciI | gccgctctagaactagtGGGCGGACGCATTGGCAACCATTCG | Construction pBM13 |
| D86 | CgATH1-t_Fw | TCctcgagggggggcccggtaccAACTGGGGATCTAGCTCA | Construction pBM13 |
| D87 | CgATH1-t_Rv_EciI | aaagggaacaaaagctgGGCGGAGTCTATGGACGCTCAACT | Construction pBM13 |
| D88 | CgNTH1-ORF_Fw_EciI | ggccgctctagaactagtGGCGGACTAACTATGAGTCAGCAACTAC | Construction pBM14 |
| D89 | CgNTH1-mCherry_Rv | GCCCTTGCTCACCATTAGGATCCcAAGACCATATATGCTCTTTTCATC | Construction pBM14 |
| D90 | CgNTH1-t_Fw | TCctcgagggggggcccggtaccATTCCAGATGATATGTAGTG | Construction pBM14 |
| D91 | CgNTH1-t_Rv_EciI | ctaaagggaacaaaagctgGGCGGAAGATTGGAAGTCATTTCTG | Construction pBM14 |
| D92 | CgNTH2-ORF_Fw_EciI | ggccgctctagaactagtGGCGGAAGCGTACGTCTTATGAGTCC | Construction pBM15 |
| D93 | CgNTH2-mCherry_Rv | GCCCTTGCTCACCATTAGGATCCcTAGGCCATAACGTGCTTTTTC | Construction pBM15 |
| D94 | CgNTH2-t_Fw | TCctcgagggggggcccggtaccATTGCTCGACGTTTTTAG | Construction pBM15 |
| D95 | CgNTH2-t_Rv_EciI | taaagggaacaaaagctgGGCGGATGTGTTATTGTTTCTCCC | Construction pBM15 |
| B4166 |  | GGAAACAGCTATGACCATG | checking insertion pYC vectors |

**References:**

1. Schwarzmüller T, Ma B, Hiller E, Istel F, Tscherner M, Brunke S, et al. Systematic phenotyping of a large-scale *Candida glabrata* deletion collection reveals novel antifungal tolerance genes. PLoS Pathog. 2014;10(6):e1004211. Epub 2014/06/20. doi: 10.1371/journal.ppat.1004211. PubMed PMID: 24945925; PubMed Central PMCID: PMCPMC4063973.

2. Yáñez-Carrillo P, Orta-Zavalza E, Gutiérrez-Escobedo G, Patrón-Soberano A, De Las Peñas A, Castaño I. Expression vectors for C-terminal fusions with fluorescent proteins and epitope tags in *Candida glabrata*. Fungal Genet Biol. 2015;80:43-52. doi: 10.1016/j.fgb.2015.04.020. PubMed PMID: 25986172.

3. Cen Y, Timmermans B, Souffriau B, Thevelein JM, Van Dijck P. Comparison of genome engineering using the CRISPR-Cas9 system in *C. glabrata* wild-type and *lig4* strains. 2017;(1096-0937 (Electronic)).
