# Supplementary figures and images for: The involvement of the Candida glabrata trehalase enzymes in stress resistance and gut colonization
Source: Virulence. 2020 Dec 28;12(1):329–45. doi: 10.1080/21505594.2020.1868825 (PMC7808424; doi:10.1080/21505594.2020.1868825)

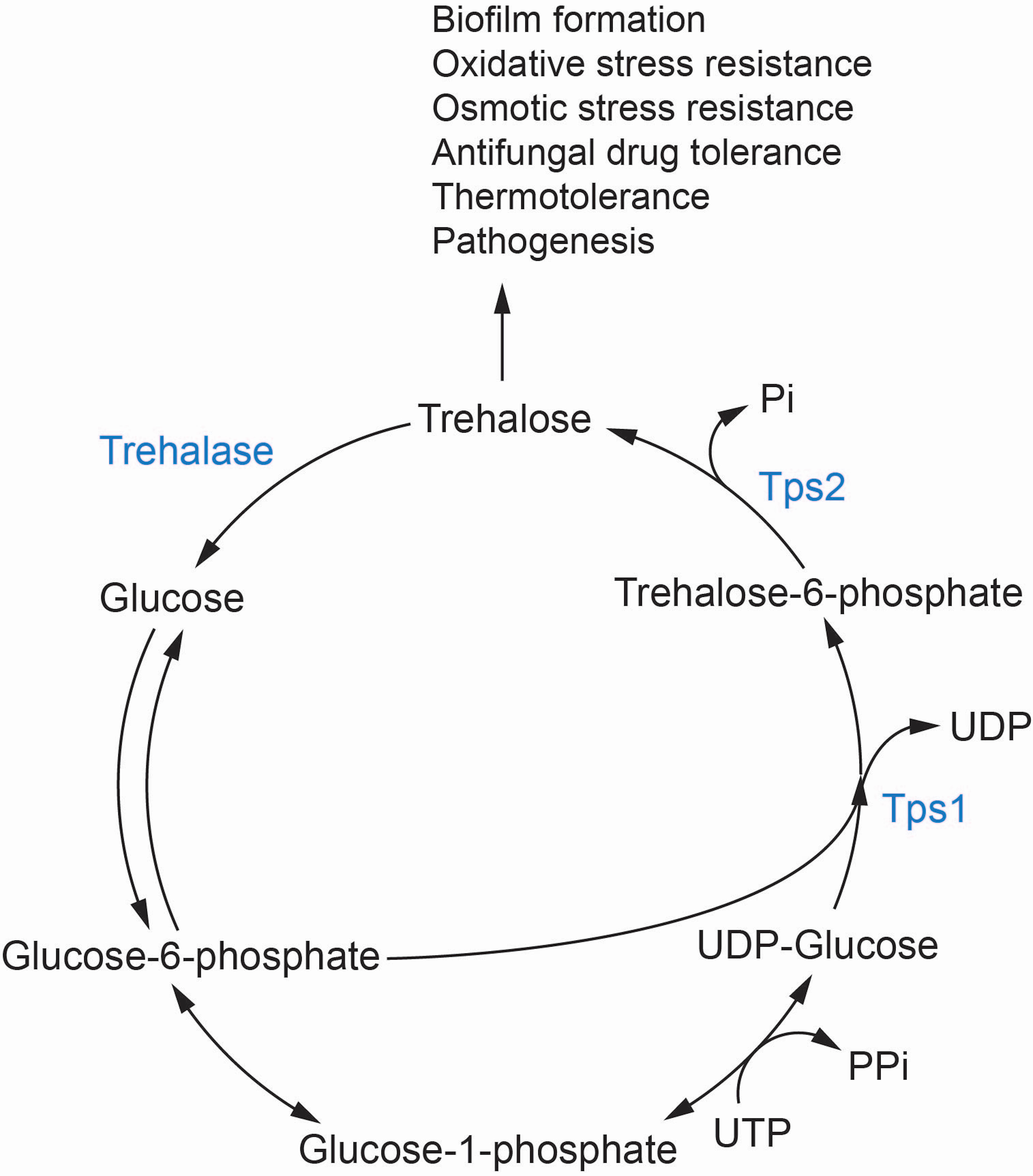

Supplement: Supplemental Material [file KVIR_A_1868825_SM6007.zip › SUPPLEMENT/Supplementary Fig S1 - trehalose pathway.jpg]

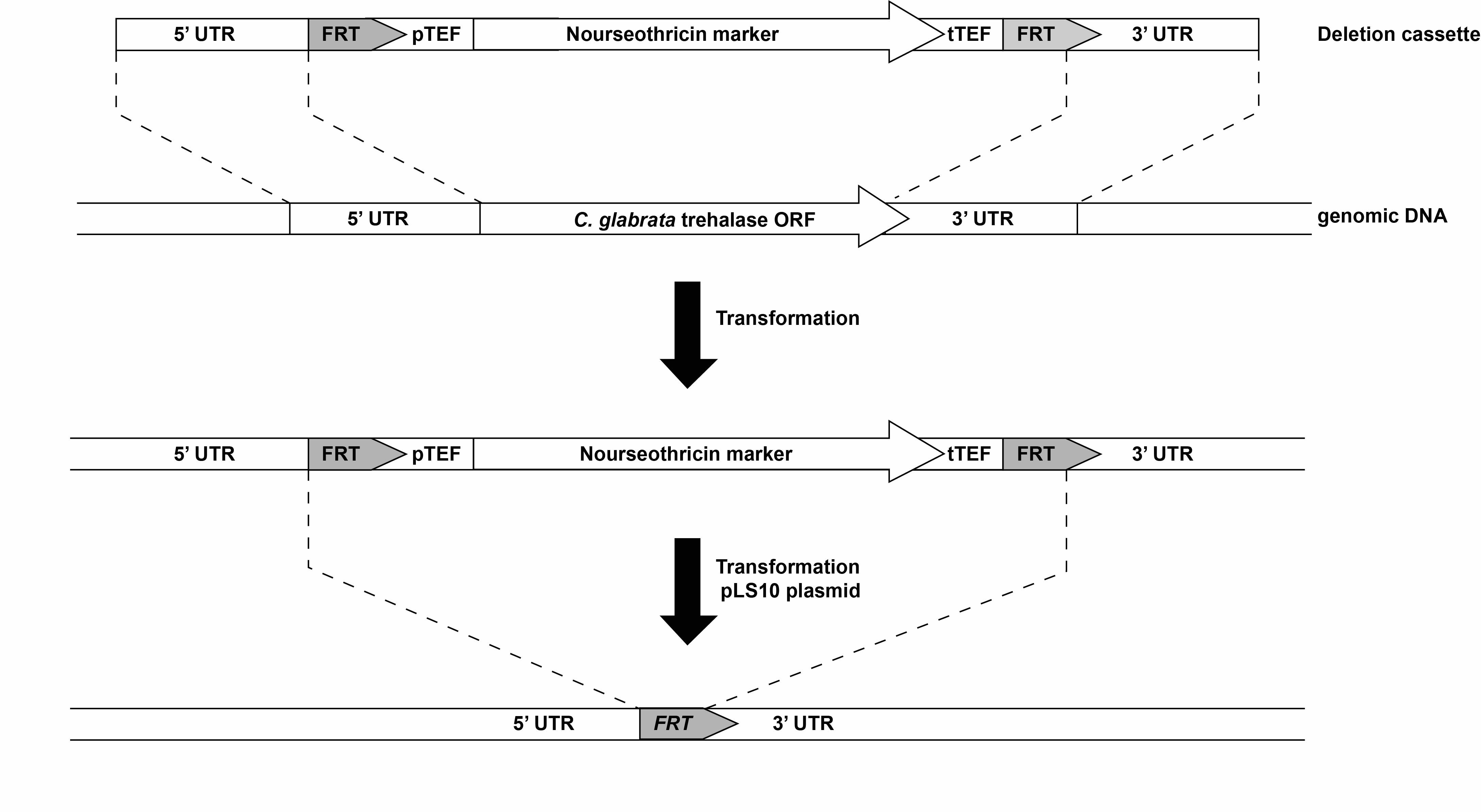

Supplement: Supplemental Material [file KVIR_A_1868825_SM6007.zip › SUPPLEMENT/Supplementary Fig S2 - Making of mutants.jpg]

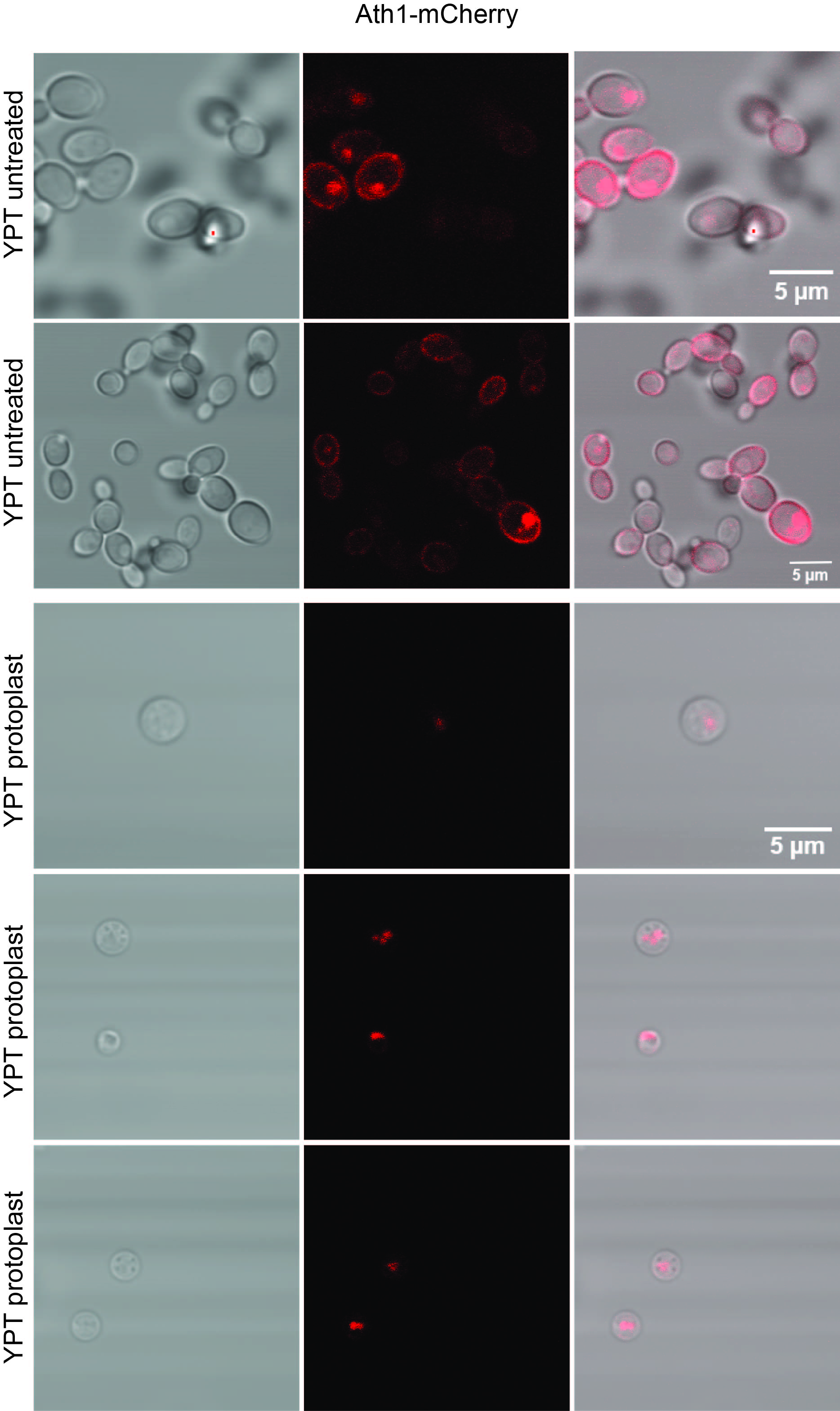

Supplement: Supplemental Material [file KVIR_A_1868825_SM6007.zip › SUPPLEMENT/Supplementary Fig S3 Microscopy overnight treO protoplast.jpg]

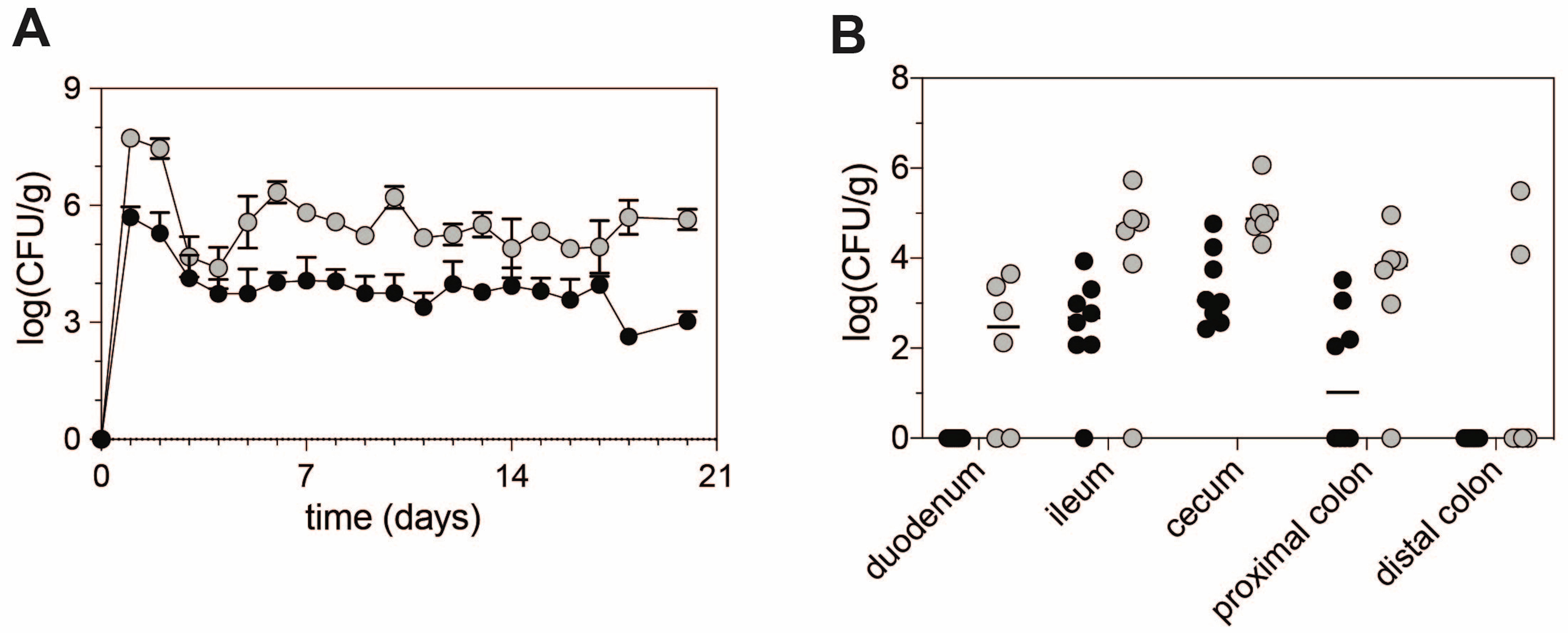

Supplement: Supplemental Material [file KVIR_A_1868825_SM6007.zip › SUPPLEMENT/Supplementary Fig S4 - GI optimisation.jpg]

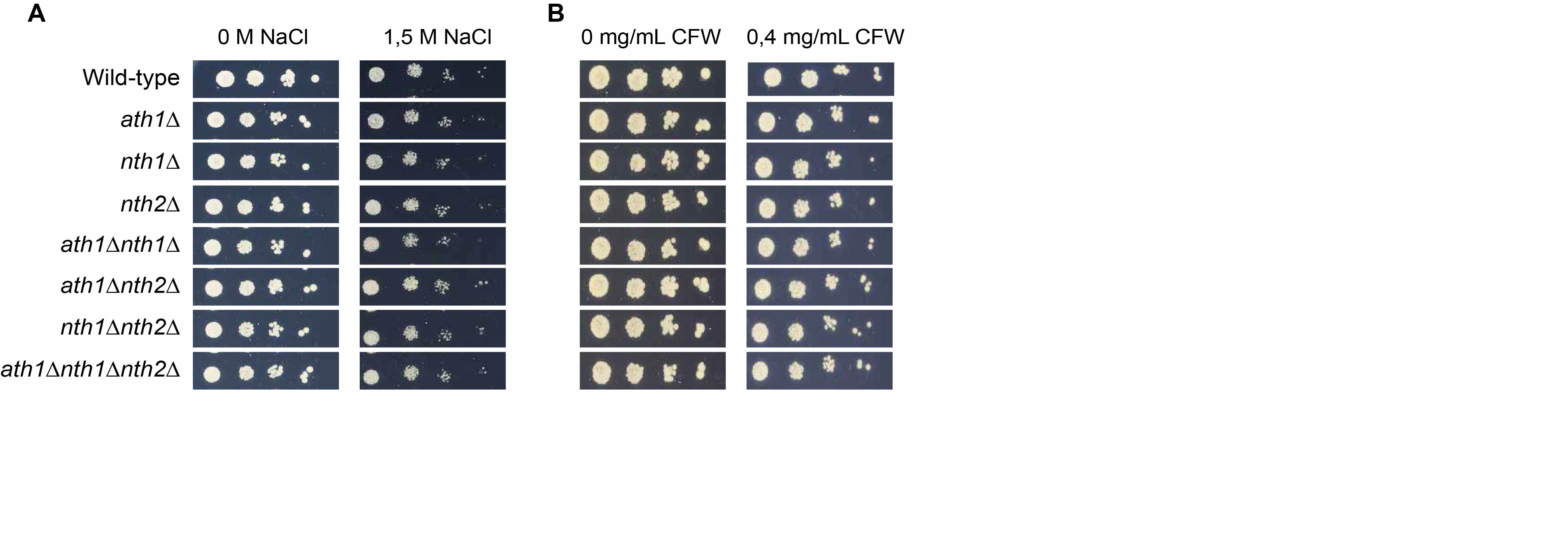

Supplement: Supplemental Material [file KVIR_A_1868825_SM6007.zip › SUPPLEMENT/Supplementary Fig S5 - spot.jpg]

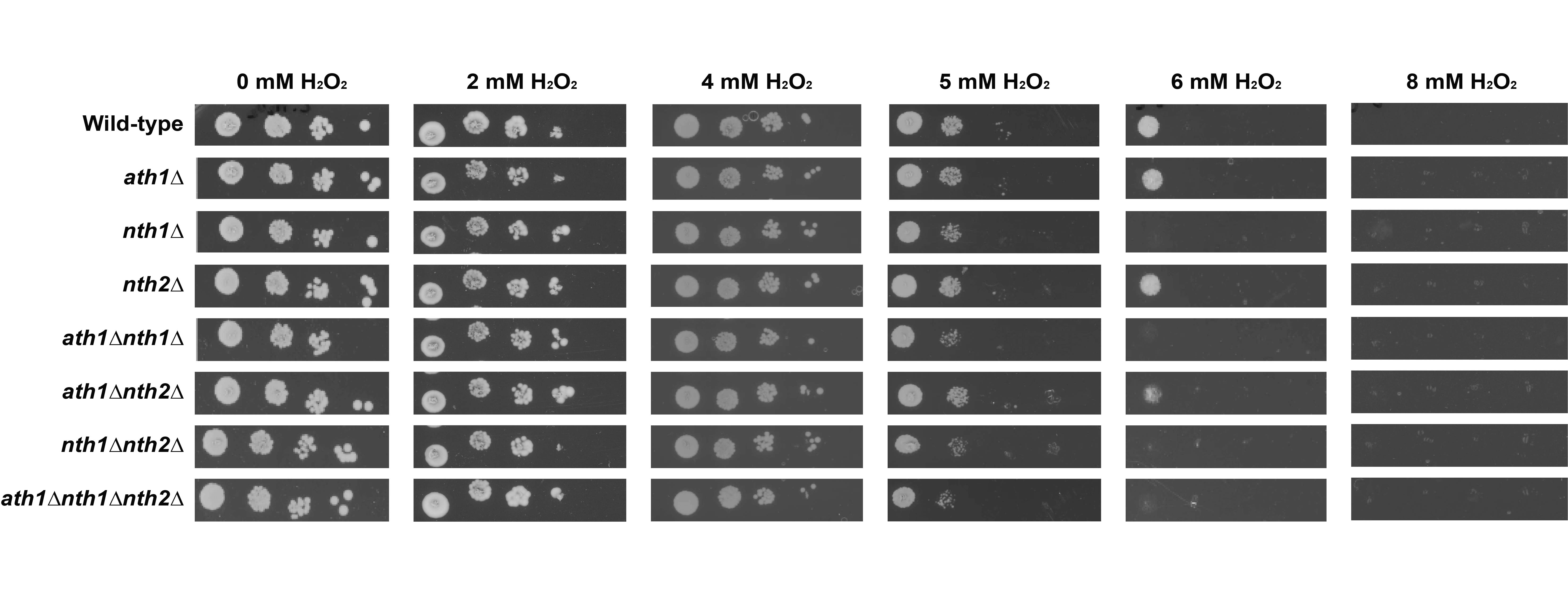

Supplement: Supplemental Material [file KVIR_A_1868825_SM6007.zip › SUPPLEMENT/Supplementary Fig S6 new.jpg]

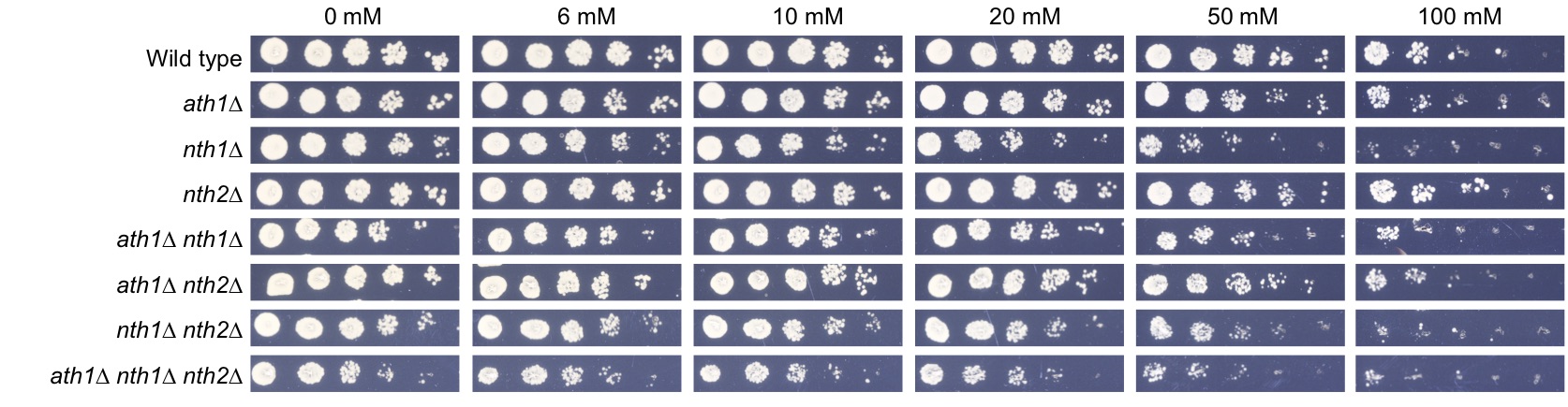

Supplement: Supplemental Material [file KVIR_A_1868825_SM6007.zip › SUPPLEMENT/Supplementary Fig S7 - survival acute H2O2 stress.jpg]

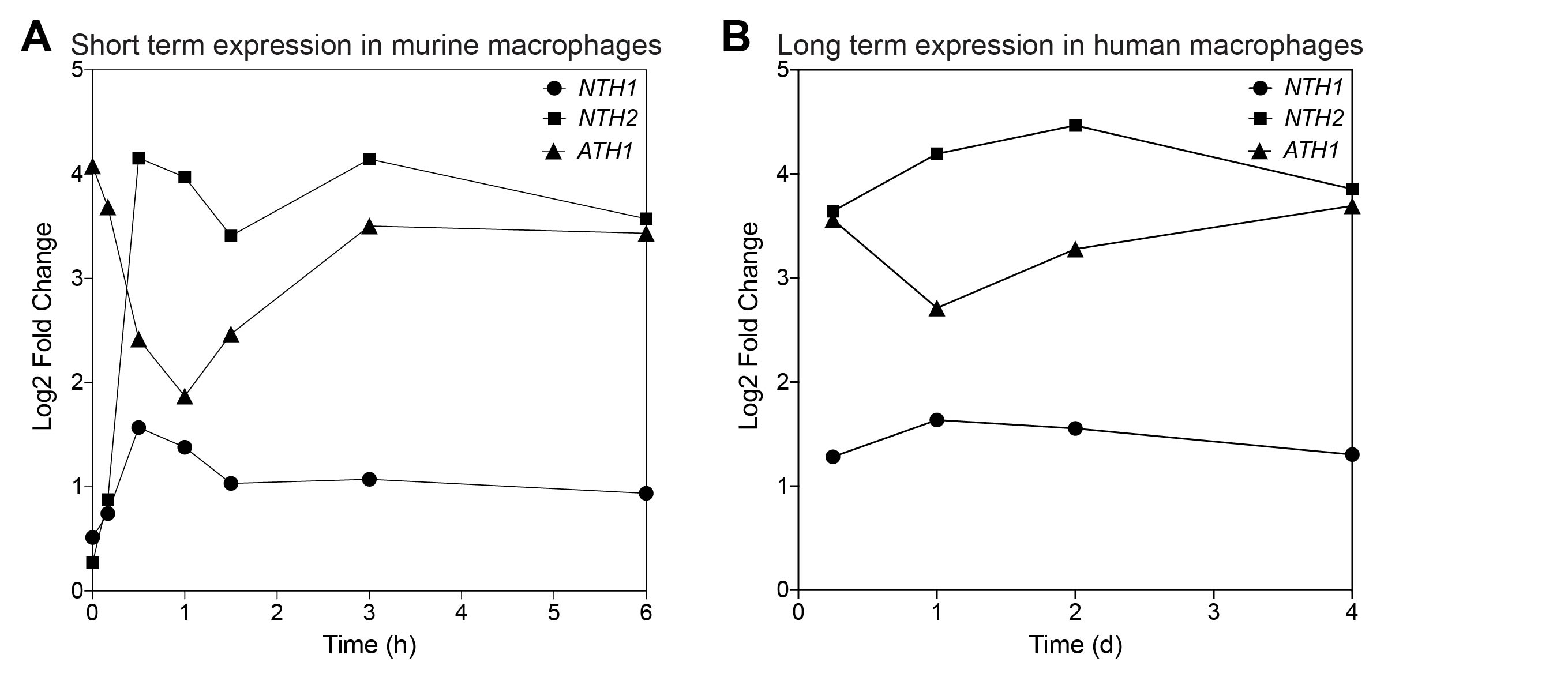

Supplement: Supplemental Material [file KVIR_A_1868825_SM6007.zip › SUPPLEMENT/Supplementary Fig S8 - expression macrophages.jpg]

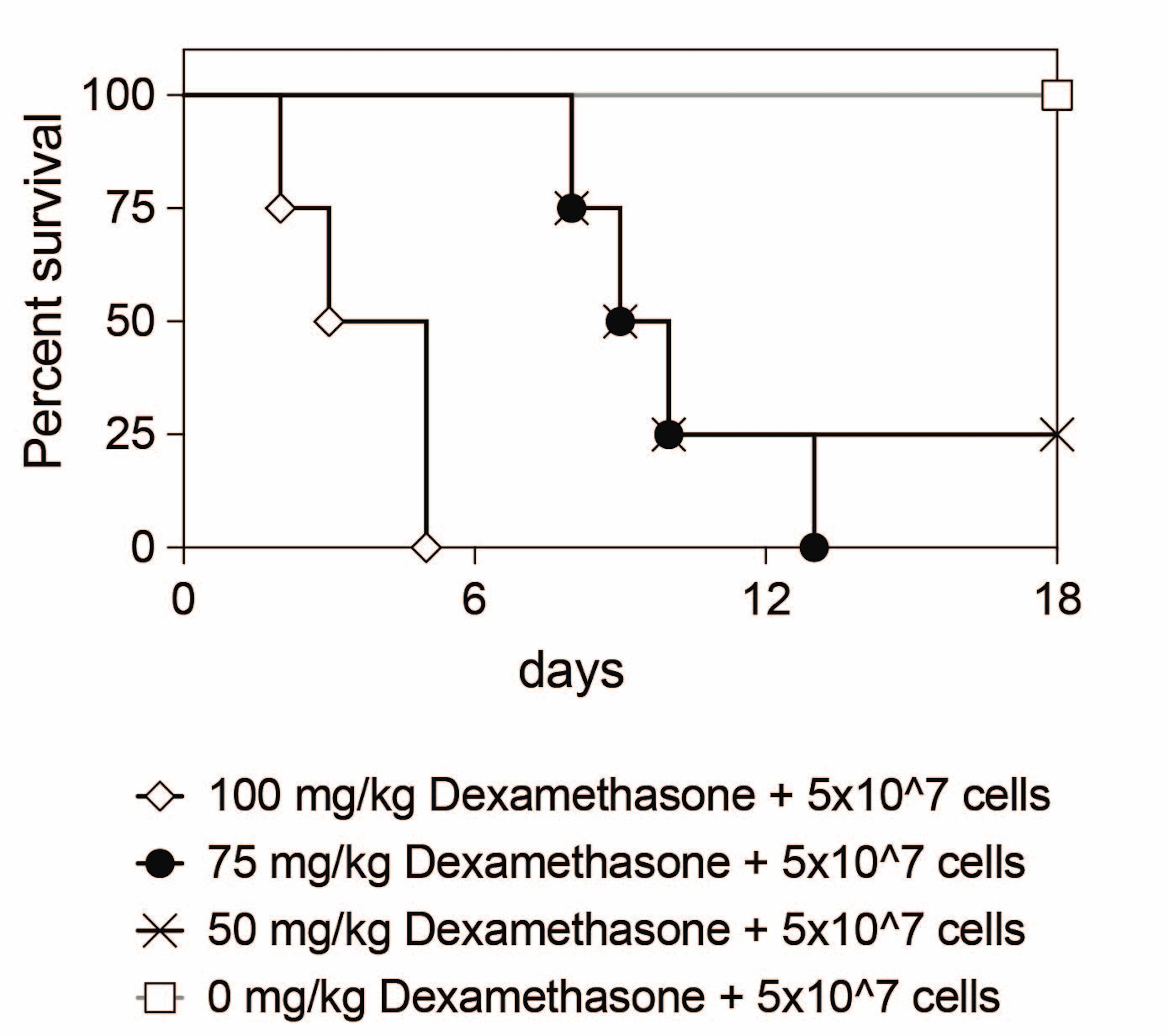

Supplement: Supplemental Material [file KVIR_A_1868825_SM6007.zip › SUPPLEMENT/Supplementary Fig S9 - survival optimization.jpg]
